# Unraveling host-pathogen dynamics in a murine Model of septic peritonitis induced by vancomycin-resistant *Enterococcus faecium*

**DOI:** 10.1080/21505594.2024.2367659

**Published:** 2024-07-01

**Authors:** Xin Yuan, Xiaolin Song, Xi Zhang, Lingfei Hu, Dongsheng Zhou, Jianlong Zhang, Chenxi Dai

**Affiliations:** a School of Life Sciences, Ludong University, Yantai, China; b State Key Laboratory of Pathogen and Biosecurity, Beijing Institute of Microbiology and Epidemiology, Beijing, China

**Keywords:** vancomycin-resistant *Enterococcus faecium*, septic peritonitis, cytokine-mediated signalling pathway, hypothermia, mathematical model

## Abstract

Vancomycin-resistant *Enterococcus faecium* (*E. faecium*) infection is associated with higher mortality rates. Previous studies have emphasized the importance of innate immune cells and signalling pathways in clearing *E. faecium*, but a comprehensive analysis of host-pathogen interactions is lacking. Here, we investigated the interplay of host and *E. faecium* in a murine model of septic peritonitis. Following injection with a sublethal dose, we observed significantly increased murine sepsis score and histological score, decreased weight and bacterial burden, neutrophils and macrophages infiltration, and comprehensive activation of cytokine-mediated signalling pathway. In mice receiving a lethal dose, hypothermia significantly improved survival, reduced bacterial burden, cytokines, and CD86 expression of MHC-II^+^ recruited macrophages compared to the normothermia group. A mathematical model constructed by observational data from 80 animals, recapitulated the host-pathogen interplay, and further verified the benefits of hypothermia. These findings indicate that *E. faecium* triggers a severe activation of cytokine-mediated signalling pathway, and hypothermia can improve outcomes by reducing bacterial burden and inflammation.

## Introduction

*Enterococcus faecium* (*E. faecium*) is a major cause of healthcare-associated infections, with nearly 50% of *E. faecium* isolates demonstrating resistance to vancomycin based on a recent national survey in Australia [1,2]. Extensive research has shown that vancomycin-resistant *E. faecium* (VRE) infection is associated to higher mortality, prolonged hospital stays, and increased treatment costs [3,4], especially in cases involving bloodstream infections [5]. Additionally, the management of significant infections often necessitates second-line antibiotic therapies, such as tigecycline and daptomycin, which may come with higher costs, reduced effectiveness, and a greater risk of toxicity compared to first-line antibiotic therapies [6,7]. Consequently, the challenges encountered in treating VRE infection have motivated researchers to delve into understanding its pathogenesis with the aim of identifying novel therapeutic targets.

The innate immune system plays a crucial role in the initial defence against invading pathogens, including *E. faecium*, by recognizing pathogen-associated molecular patterns (PAMPs). Leendertse and colleagues firstly discovered that MyD88, at least in part through Toll-like receptor 2 (TLR2), is essential for the effective clearance of *E. faecium* during peritonitis by facilitating neutrophil recruitment to the infection site [8,9]. They also identified the importance of peritoneal macrophages and neutrophil attraction to the primary site of infection in the clearance of *E. faecium* and subsequent regulation of systemic inflammatory response [9–11]. Additionally, they revealed that complement deficiency severely impairs the clearance of peritonitis and subsequent systemic infection [9,12]. Another study demonstrated that *E. faecium* secreted-peptidoglycan hydrolase (SagA) can activate nucleotide-binding oligomerization domain-containing protein 2 (NOD2) host immune pathways and enhance tolerance to pathogens [13]. These findings provide a solid foundation for understanding the mechanisms of *E. faecium* infection, but a comprehensive investigation of host-pathogen interactions has not been explored using emerging approaches such as transcriptomics and mathematical modelling.

In this study, we utilized a VRE strain to establish a murine model of septic peritonitis. Through time-course analysis of bacterial burden, histological examination, RNA sequencing (RNA-seq), cytokines detection, and flow cytometry, we uncovered dynamic changes in the host peritoneal cavity at a sublethal dose. We observed that the cytokine-mediated signalling pathway was significantly upregulated, and MHC-II^+^ recruited macrophages remarkably increased the expression of CD86. Subsequently, we evaluated the protective effects of therapeutic hypothermia, a potential anti-inflammatory treatment [14–16], against a lethal dose of VRE infection. Finally, using the time-course data obtained from biological experiments involving 80 animals, we developed a mathematical model to characterize the interplay between various immune cells and bacteria. Model simulation revealed that the benefits of hypothermia could be attributed to the significant downregulation of two key parameters, namely the bacterial growth rate and the transfer rate from M0 (resting state of macrophages) to M1 (classically activated macrophages) of recruited macrophages.

## Results

### *E. faecium* infection caused severe septic peritonitis

**Figure 1.**
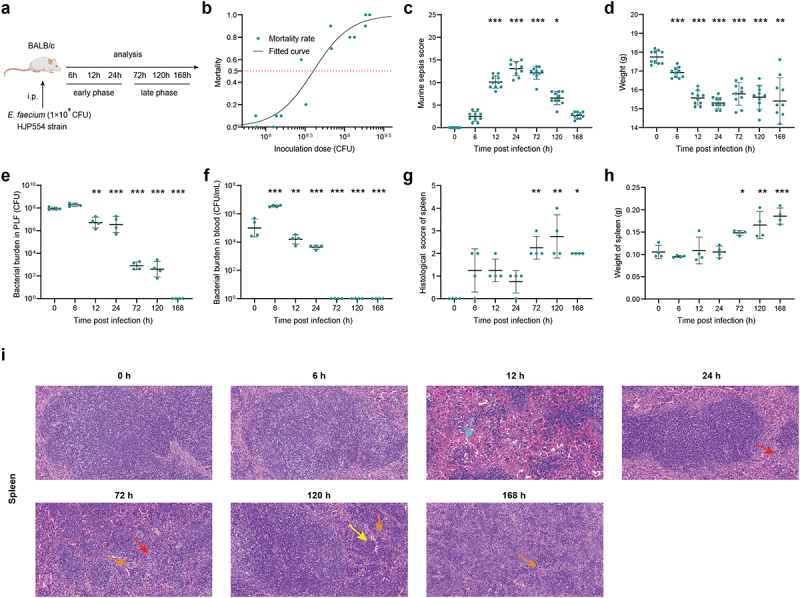
*E.* *faecium* infection causes severe septic peritonitis. (a) Scheme showing the experimental schedulefor *E.* *faecium* infection. (b) Mortality after *E.* *faecium* at different doses, n = 10. (c) Murine sepsis score after *E.* *faecium* at the sublethal dose. (d) Mice weight change after *E.* *faecium* at the sublethal dose. (e–f) Bacterial burden in PLF and blood. (g) Histological score of spleen. (h) spleen weight. (i) Histological analysis of spleen, liver and kidney by hematoxylin and eosin (H&E) staining. Red arrows indicate inflammatory cell infiltration, light blue arrows indicate hemorrhage, yellow arrows indicate karyorrhexis, orange arrows indicate the presence of multinucleated giant cells (original magnification ×20). *: P < 0.05, **: P < 0.01, ***: P < 0.001 when compared to 0 h.

We used an experimental model of septic peritonitis by intraperitoneal injection of *E. faecium* HJP554 strain, in BALB/c mice at various doses (Figure 1a). We determined that the sublethal dose, half lethal dose, and lethal dose were 1 × 10^8^ colony-forming units (CFU), 4 × 10^8^ CFU, and 2 × 10^9^ CFU, respectively (Figure 1b). We next adopted the sublethal dose to fully and deeply investigate the interaction mechanism of *E. faecium* with the host immune system. Time course analysis of various parameters was performed during the early phase (6 h, 12 h, 24 h) and late phase (72 h, 120 h, 168 h), with 0 h serving as negative control. In the early phase, mice developed sepsis, as indicated by the increased murine sepsis score (Figure 1c) and decreased weight (Figure 1d). It took until 168 h for the mice to fully recover from the septic symptoms and return to their pre-disease state (Figure 1c). The initial bacterial burden in peritoneal lavage fluid (PLF) after inoculation was approximately 1 × 10^8^ CFU, and peaked at around 1.8 × 10^8^ CFU at 6 h (Figure 1e). More than 95% of the bacteria were cleared within 24 h, and infection was resolved by 168 h (Figure 1e,f). The bacterial burden of blood, spleen, liver, kidney, heart, and lung showed a similar trend to that in PLF (Figure 1e, Figure S1a-e). Significant histopathological changes occurred at 72 h post-infection and continued until 168 h (Figure 1gi, Figure S1f-i). In addition, there was a remarkable increase in spleen weight, gradually amplifying over time, partially suggesting the activation of adaptive immune response (Figure 1h).

### RNA-seq analysis revealed the peritoneal inflammation after *E. faecium* infection

**Figure 2.**
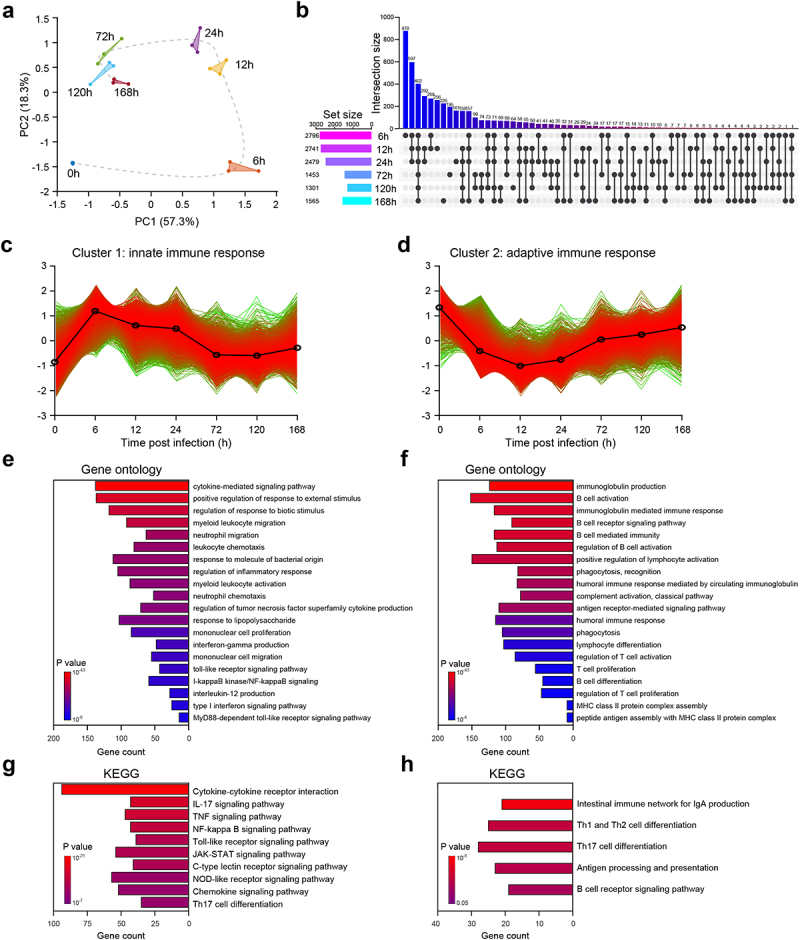
RNA-seq analysis reveals the mechanism of host immune response after *E.* *faecium* infection. (a) Principal component analysis results of RNA-seq data. The same color represents replicates of the same stage. (b) Histograms show differential expressed genes at different time points. (c–d) Cluster 1 and 2 of gene expression identified by Mfuzz cluster analysis. (e–f) Gene ontology enrichment analysis revealed the biological processes of cluster 1 and 2. (g–h) KEGG enrichment analysis of cluster 1 and 2.

To investigate transcriptional changes, we collected and sequenced RNA from cell suspensions of PLF infected with *E. faecium*. We aimed to analyse the dynamics of gene expression profiles before and during infection. We employed principal component analysis to assess the quality of data and the distribution of samples (Figure 2a). Our analysis revealed that the first principal component (PC1) accounted for 38.8% of the total expression differences, while the second principal component (PC2) accounted for 22%. Additionally, we observed excellent reproducibility among replicate samples from the same time point, affirming the consistency of our experimental procedures. However, samples collected at different time points exhibited distinctive transcriptional characteristics, indicating temporal changes in gene expression patterns during infection.

Compared with 0 h when mice are uninfected, we found that the number of differentially expressed genes (DEGs) at 6 h, 12 h, 24 h, 72 h, 120 h and 168 h were 5681, 5786, 5408, 3313, 2962 and 2989, respectively (Figure S2a). From Figure 2b, we observed that at 6 h post-infection, there was the highest number of unique upregulated DEGs with a total of 878, suggesting sharp and distinct changes in host gene expression (Figure 2b). Additionally, at 6 h, there were 432 upregulated DEGs that were intersected with other time points, indicating a shared pattern of gene expression changes (Figure 2b). The number of DEGs decreased over time, suggesting that the host has a repair process after septic peritonitis (Figure S2a). Consistent with previous studies [8,13], we observed a significant increase in the expression of *Tlr2*, *Myd88* and *Nod2* after infection (Figure S2b), indicating that both types of *E. faecium* can activate these signalling pathways. However, in contrast to a previous study [8], we also found an increased expression of *Tlr4* (Figure S2b).

To investigate the temporal changes in gene expression levels, we categorized the DEGs into four gene clusters based on their expression patterns using a clustering algorithm called Mfuzz. Subsequently, we performed a Gene Ontology (GO) enrichment analysis to evaluate the functional processes associated with each gene cluster (Figure 2c-f, Figure S4). These gene clusters captured distinct biological processes that are relevant to the progression of the disease. We have assigned the following names to the four gene clusters based on the results of the GO enrichment analysis: cluster 1, innate immune response; cluster 2, adaptive immune response; cluster 3, autophagy and metabolic processes; and cluster 4, energy and metabolic processes.

DEGs of cluster 1 reached their highest expression level at 6 h after infection, followed by a continuous decrease (Figure 2c). The GO enrichment analysis indicated that cluster 1 was primarily involved in biological processes related to innate immune response. These processes include cytokine-mediated signalling pathway, positive regulation of response to external stimulus, regulation of response to biotic stimulus, myeloid leukocyte migration, neutrophil migration, etc (Figure 2e). The Kyoto Encyclopedia of Genes and Genomes (KEGG) analysis indicated a comprehensive activation of cytokine-mediated signalling pathways (Figure 2g, Figure S3), suggesting that the mice experienced a severe cytokine storm. Furthermore, *E. faecium* activated a number of innate immune signalling pathways, including IL-17, TNF, NF-kappa B, Toll-like receptor, JAK-STAT, C-type lectin receptor, NOD-like receptor Chemokine, etc (Figure 2g). Such activation is often associated with an exaggerated immune response and this information can provide valuable insights into potential therapeutic targets for further investigation.

In cluster 2, the gene expression decreased immediately after infection, reaching its lowest point at 12 h post-infection, and then slowly started to increase again (see Figure 2d). The GO analysis indicated that cluster 2 was primarily involved in biological processes related to the adaptive immune response, highlighting its crucial role in the activation and regulation of the adaptive immune response during the infection. These processes included immunoglobulin production, B cell activation, immunoglobulin-mediated immune response, regulation of T-cell activation, T-cell proliferation, etc (Figure 2f). KEGG enrichment analysis further validated above results, as these signalling pathways were activated, including intestinal immune network for IgA production, Th1 and Th2 cell differentiation, Th17 cell differentiation, antigen processing and presentation, B cell receptor and etc (Figure 2h). For clusters 3 and 4, the gene expression also decreased after infection and then gradually increased again (Figure S4ab). The GO and KEGG enrichment analysis both revealed that clusters 3 and 4 were primarily involved in biological processes related to the adaptive immune response, autophagy, energy, and metabolic processes (Figure S4c-f).

### Significantly increased expression of cytokines levels further validated the inflammatory state after *E. faecium* infection

**Figure 3.**
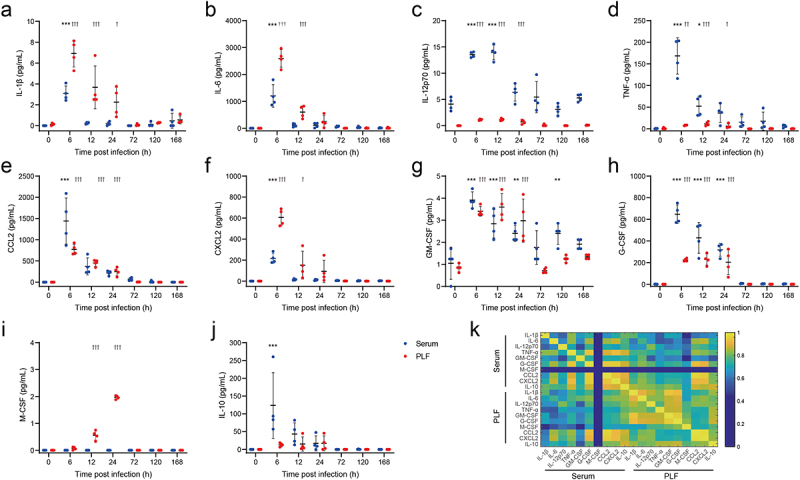
Expression of cytokines significantly increased after *E. faecium* infection.(a) IL-1β, (b) IL-6, (c) IL-12p70, (d) TNF-α, (e) CCL2, (f) CXCL2, (g) GM-CSF, (h) G-CSF, (i) M-CSF, (j) IL-10, (k) Correlation analysis among above cytokines. PLF: peritoneal lavaguids. *: *P* < 0.05, **: *P* < 0.01, ***:  P< 0.001 when compared to 0 h in serum. †: *P* < 0.05, ††:  P< 0.01, †††:  P< 0.001 when compared to 0 h in PLF.

RNA-seq analysis revealed a comprehensive activation of cytokine-mediated signalling pathways following infection. Therefore, four proinflammatory cytokines (IL-1β, IL-6, IL-12p70, and TNF-α), two chemokines (CCL2 and CXCL2), three colony-stimulating factors (GM-CSF, G-CSF, and M-CSF), and an anti-inflammatory cytokine (IL-10) were measured in both PLF and serum. In comparison to uninfected mice, the levels of IL-1β, IL-6, IL-12p70, TNF-α, CCL2, CXCL2, GM-CSF, and G-CSF were significantly increased in both PLF and serum after infection (Figure 3a-h). Notably, M-CSF showed a remarkable increase only in PLF (Figure 3i), while IL-10 exhibited a notable increase only in serum after infection (Figure 3j). Correlation analysis indicated a significant correlation among these cytokines, chemokines, and colony-stimulating factors (Figure 3k), except for M-CSF in PLF. These findings suggested an immediate inflammatory response following infection, accompanied by the proliferation, differentiation, recruitment, and functional activation of monocytes, macrophages and neutrophils [17–19].

### *E. faecium* infection induced sharp changes of innate immune cells in PLF

**Figure 4.**
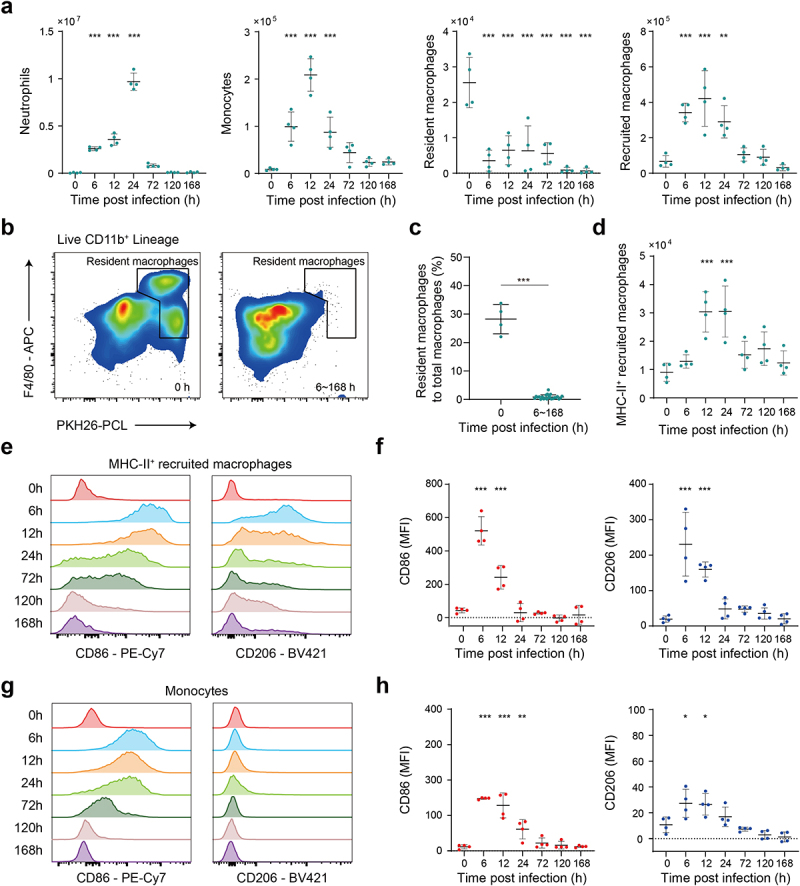
Flow cytometry analysis reveals the response of innate immune cells after *E.* *faecium* infection. (a) Number of innate immune cells, including neutrophils, monocytes, resident macrophages and recruited macrophages before and after infection. (b) Number of resident macrophages was significantly decreased after infection. (c) The percentage of resident macrophages to total macrophages was significantly decreased after infection. (d) Number of MHC-II^+^ recruited macrophages before and after infection. (e) CD86 and CD206 expression on MHC-II^+^ recruited macrophages. (f) The MFI of CD86 and CD206 expression on MHC-II^+^ recruited macrophages. (g) CD86 and CD206 expression on monocytes. (h) The MFI of CD86 and CD206 expression on monocytes. MFI: mean fluorescence intensity. *: P < 0.05, **: P < 0.01, ***: P < 0.001 when compared to 0 h.

We next employed flow cytometry to accurately analyse the response of immune cells before and after *E. faecium* infection (see gating strategy in Figure S5a). To specifically label peritoneal phagocytes, we injected fluorescent PKH26-PCL dye intraperitoneally 24 h prior to infection. Neutrophils (Ly6G^+^), monocytes (PKH26-PCL^Lo^ Ly6C^+^), recruited macrophages (PKH26-PCL^Lo^ Ly6C^−^ F4/80^+^) were promptly recruited after infection (Figure 4a), which was paralleled by a reduction in resident macrophages (PKH26-PCL^Hi^ F4/80^+^), a phenomenon commonly known as the macrophage disappearance reaction (Figure 4b) [20]. By 72 h post infection, neutrophils, monocytes and recruited macrophages were largely cleared. Throughout the entire post infection period, macrophages predominantly exhibited a PKH26-PCL^Lo^ phenotype (Figure 4c). We also observed that MHC-II^+^ recruited macrophages may play crucial role in the immune process, as they displayed characteristics of both proinflammatory M1-type and anti-inflammatory M2-type (alternatively activated macrophages) (Figure 4d–f). The mean fluorescence intensity (MFI) of CD86 and CD206 expression on MHC-II^+^ recruited macrophages both peaked at 6 h and then steadily decreased until 24 h post infection (Figure 4e,f). Furthermore, we observed the proinflammatory M1-type activation in monocytes (Figure 4g,h), with a significant increase in the MFI of CD86 expression at 6 h, 12 h, and 24 h post-infection.

### Hypothermia improved survival through anti-inflammation and anti-infection effects

**Figure 5.**
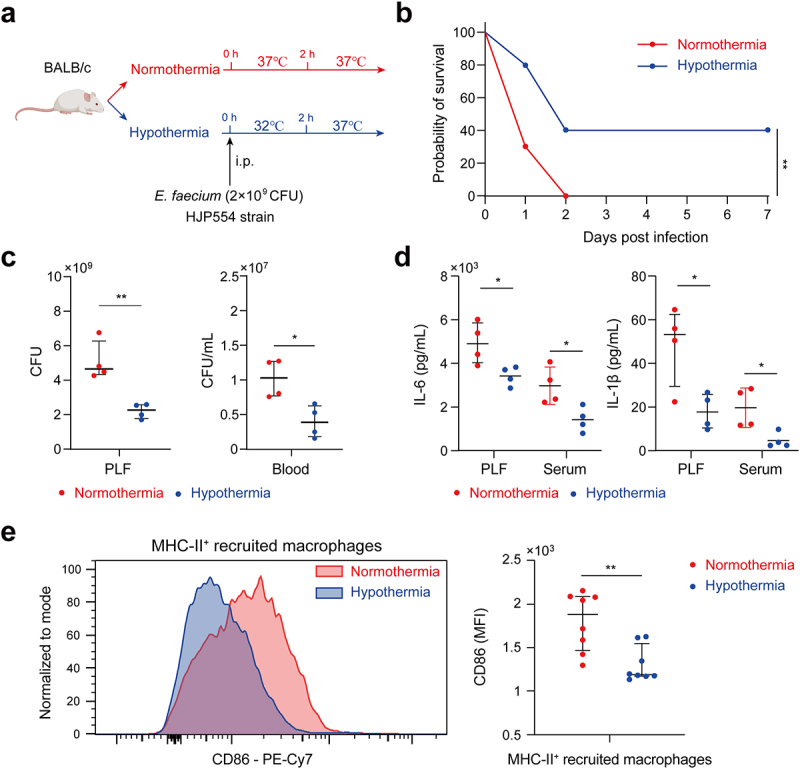
Hypothermia was associated with survival. (a) Hypothermia protocol. (b) Survival of hypothermia and normothermia-treated mice after *E.* *faecium* infection; n = 10. (c) Bacterial burden of PLF and blood at 6 h after *E.* *faecium* infection. (d) IL-6 and IL-1β in PLF and serum at 6 h after *E. faecium* infection. (e) CD86 expression of MHC-II^+^ recruited macrophages at 6 h after *E.* *faecium* infection. Blue represents hypothermia and red represents normothermia *: P < 0.05, **: P < 0.01.

On the basis of animal evidence and human studies, hypothermia has been used as a treatment of serious infections for decades by reducing inflammation, minimizing sepsis-related damage to organs and limiting the dissemination of infection [14–16]. In this study, we aimed to investigate whether hypothermia could improve the survival of mice infected by *E. faecium* with a lethal dose of 2 × 10^9^ CFU (Figure 5a). The results demonstrated a significantly higher survival rate in the hypothermia group compared to the normothermia group (Figure 5b). Furthermore, hypothermia resulted in a notable decrease in the bacterial burden in both the PLF and blood at 6 h after infection (Figure 5c). Levels of pro-inflammatory cytokines IL-1β and IL-6 were reduced in the hypothermia group at 6 h post-infection (Figure 5d). Flow cytometry analysis of the peritoneal cavity revealed a significant reduction in CD86 expression on MHC-II^+^ recruited macrophages in the hypothermia group compared to the normothermia group (Figure 5e). These findings suggest that hypothermia could be a beneficial treatment for mice infected with *E. faecium*.

### Mathematical model revealed the dynamics of host-pathogen interaction and benefits of hypothermia in *E. faecium* infection

**Figure 6.**
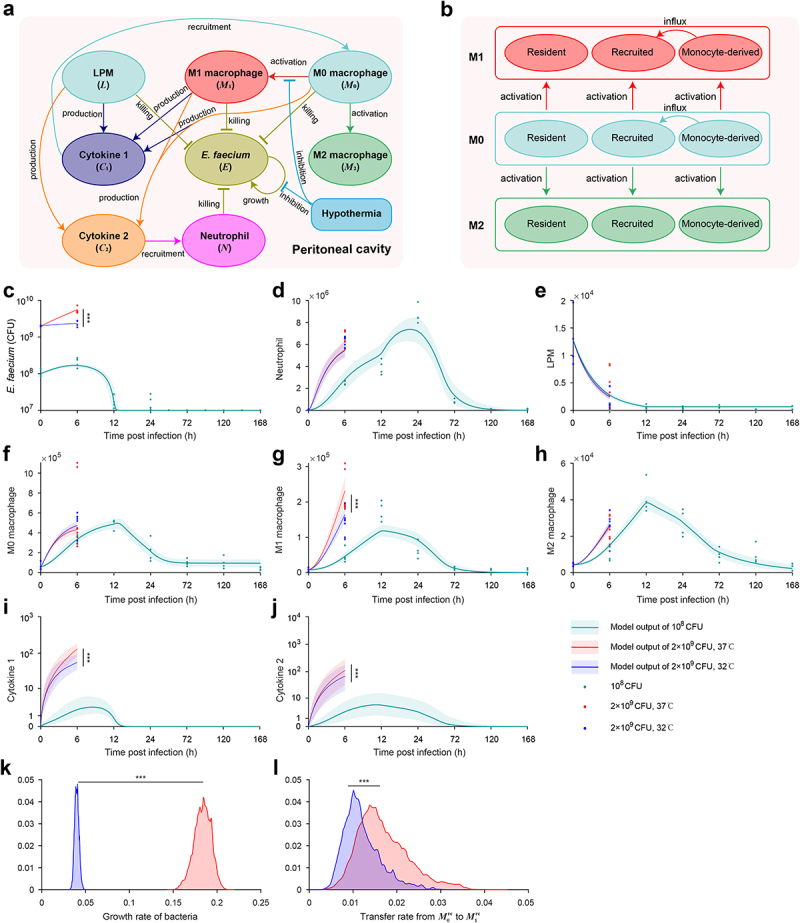
Mathematical model revealed the interaction of immune cells and *E.* *faecium* in the peritoneal cavity. (a) Model schematic. *E.* *faecium* can grow exponentially in the absence of immune cells. It can be eliminated by LPM, M0, M1, and neutrophils. M0 macrophages can differentiate into M1 and M2 macrophages. M0, M1, and LPM secrete cytokines to recruit macrophages and neutrophils. (b) The interactions among macrophages from different source. Monocyte-derived macrophages can influx to recruited population. Model estimates of *E.* *faecium* (c), neutrophils (d), LPMs (e), M0 macrophages (f), M1 macrophages (g), M2 macrophages (h), cytokine 1 (i), cytokine 2 (j). The two key parameters determining the beneficial effects of hypothermia is growth rate of bacteria (k) and transfer rate from M0 to M1 in recruited macrophages. Cytokine 1: cytokines for macrophage recruitment. Cytokine 2: cytokines for neutrophil recruitment. LPM, large peritoneal macrophage. ***: P < 0.001.

In this section, we have developed a computational model using ordinary differential equations (ODEs) that comprehensively describes the dynamic interaction among immune cells, cytokines, and bacteria during *E. faecium* infection (see details in section S1 in supplementary information). Additionally, we elucidated the impact of sublethal and lethal inoculation doses on the immune response and showed the beneficial effects of hypothermia. This model is based on previous studies as well as the observed experimental data and phenomena in our present study [21,22]. It incorporates various essential processes, including pathogen growth and elimination, the disappearance of large peritoneal macrophages (LPMs), macrophage polarization into phenotypes M1 and M2, interactions among macrophages from different sources, cytokines release, and neutrophil recruitment. System variables include cell populations given by *M*_0_ (M0 macrophages), *M*_1_ (M1 macrophages), *M*_2_ (M2 macrophages), *L* (large peritoneal macrophages), *N* (neutrophils), *C*_1_ (cytokines for macrophage recruitment), *C*_2_ (cytokines for neutrophil recruitment), as well as *E* (*E. faecium*) (Figure 6a, Table S2). Then, according to cell markers of PKH26-PCL and Ly6C, macrophages were further divided into three subsets, resident, recruited and monocyte-derived cells (Figure 6b, Table S2).

After fitting the model to experimental data (see details in supplementary information), we first found that the model accurately recapitulates the changes in all system variables observed in the animal experiments under sublethal doses of *E. faecium* challenge, including pathogen growth and elimination (Figure 6c), neutrophil infiltration (Figure 6d), the disappearance of LPMs (Figure 6e), macrophage polarization into phenotypes M1 and M2 (Figure 6f-h), and cytokines release (Figure 6i,j) and the interactions among macrophages from different sources (Figure S6).

Next, we demonstrated the beneficial effects of hypothermia under lethal doses. We found that the model outputs are consistent with the experimental results. In comparison to the normothermia group, the hypothermia group exhibited a significantly slower growth rate of bacteria (Figure 6c), a remarkable decrease in the number of M1 macrophages (Figure 6g), and a notable reduction in the secretion of pro-inflammatory cytokines (Figure 6i,j). Further analysis revealed that the beneficial effects of hypothermia were attributed to significant changes in two critical parameters in the mathematical model, namely the bacterial growth rate (Figure 6k, Table S3) and the transfer rate from M0 to M1 of recruited macrophages (Figure 6l, Table S3).

## Discussion

In the present study, based on the time course analysis of murine sepsis score, histological examination, bacterial burden, cytokines, and immune cells, we comprehensively unravelled the interplay of host immune system and *E. faecium* and found the severe activation of cytokine-mediated signalling pathway. Then in mice inoculated with a lethal dose, hypothermia exerted protective effects by decreased bacterial burden, cytokine levels, and CD86 expression of MHC-II^+^ recruited macrophages. Finally, we constructed a mathematical model to recapitulate the interplay of immune cells and bacteria, and verified the benefits of hypothermia.

In order to investigate the immune response to *E. faecium* septic peritonitis, a model was established where a peritoneal infection was induced in healthy mice, from which they were able to recover. Injecting 2 × 10^9^ CFU *E. faecium* resulted in lethality in all mice, while 10^8^ CFU caused a systemic infection that was eventually cleared (Figure 1b). Previous study reported that the lethal dose of the bacteria was more than 10^10^ CFU [8], indicating a higher virulence of our *E. faecium*. Phenotypically, within 6 h of infection, mice showed a decrease in body weight accompanied by an increase in bacterial burden. The murine sepsis score began to increase 6 hours post-infection. At 24 h post-infection, mice displayed the most severe symptoms with the lowest body weight and highest murine sepsis score, and then their body weight and symptoms gradually recovered (Figure 1c-d). Significant histological changes occurred 72 h post-infection, mainly in the spleen, liver, kidney, and lung (Figure 1g, Figure S1f-i), which is different from previous study that 10^8^ CFU did not result in histopathological changes in any organs [8]. Bacterial burden in PLF, spleen, heart, and blood were completely cleared at 168 h after infection, while that in the liver, kidney, and lung were not completely cleared within 7 days (Figure 1e-f, Figure S1a-e). However, Leendertse et al. reported that *E. faecium* were completely cleared within 48 h^8^. Based on the results of RNA-seq, cytokine expression, and flow cytometry, the host’s antibacterial response was divided into two stages: the innate immune phase and the adaptive immune phase. The innate immune phase (6 h, 12 h, 24 h) showed significant activation of innate immune signalling pathways (cytokine-mediated signalling pathways, myeloid leukocyte migration, neutrophil migration, Figure 2e), significant secretion of pro-inflammatory cytokines (Figure 3), infiltration of neutrophils and monocytes, and recruitment of macrophages (Figure 4a). Consistent with previous studies [8,13], we found elevated expression of *Tlr2*, *Myd88*, and *Nod2* (Figure S2b). Interestingly, we also discovered significant changes in *Tlr4* expression, which differed from previous research [8]. This may be attributed to two main factors: virulence factors and additives. The *E. faecium* strain HJP554 utilized in this study has been found to harbour various virulence factors such as *sgrA*, *ptsD*, *orf1481*, *esp* and *hyl*, but strain E155 did not include *esp* and *hyl*. *hyl* can enhance colonization, and *esp* can promote biofilm formation and cell adhesion. It has been reported that *esp* contributes to the pathogenesis of *E. faecium* endocarditis [23,24]. The presence of these two virulence factors boosts bacterial virulence and triggers a more severe inflammatory response. Moreover, mice were intraperitoneally injected with a mixture comprising 40% sterile rat faecal extract and 10% sterile yeast extract. Studies have shown that adding sterile rat faecal extract can decrease LD_50_ by over 10-fold, leading to an increased inflammatory response. Similarly, sterile yeast extract offers a nutrient-rich environment for *E. faecium* proliferation, thereby boosting its virulence. These factors collectively contribute to the upregulation of *Tlr4* expression. When considering histological scores and the expression levels of these four genes (*Tlr2*, *Tlr4*, *Myd88*, *Nod2*), our model caused more severe damage than the model established by Leendertse et al. [8]. The adaptive immune phase (72 h, 120 h, 168 h) was characterized by antibody production, and activation of B cells and T cells (Figure 2f).

In the sterile peritoneal cavity, macrophage subsets have been classified, according to their phenotypes, functions, and origins, as LPMs and small peritoneal macrophages (SPMs) [25]. In a peritoneal infection of *E. coli*, the authors found that LPMs disappeared from the peritoneal cavity and then drove the formation of mesothelium-bound, fibrin-dependent, multicellular aggregates, which enable peritoneal immune cells to control bacterial infection [20]. However, unlike sterile inflammation where resident macrophages reappear at 3 d post-infection [26], we did not observe this phenomenon until the end of the observation period, which could potentially be attributed to the more severe inflammation caused by the bacterial infection compared to zymosan A. We categorized macrophages into three groups: resident, recruited, and monocyte-derived, based on cell markers F4/80, PKH26-PCL, and Ly6C. While resident macrophages disappear entirely within 6 h after infection, the other two groups persist for most of the observation period. Notably, MHC-II^+^ recruited macrophages, displaying distinct M1-like phenotype, may play a critical role in antibacterial response (Figure 4g,f). Furthermore, M-CSF, known as a macrophage colony-stimulating factor, exhibited a significant increase only in PLF (Figure 3i). M-CSF initially triggers the differentiation of myeloid cells into monocyte precursors and subsequently facilitates the differentiation of monocytes into macrophages [27]. This further confirms the rise in recruited monocyte-derived macrophages as observed by flow cytometry.

Considering the notable inflammatory response, we tested the protective effect of hypothermia. Previous studies reported that hypothermia increased survival duration during experimental sepsis by anti-inflammatory effects, energy metabolism, and oxidative responses [14,28]. Consistent with these studies, our present study revealed that hypothermia manifests beneficial effects in various aspects: inhibiting bacterial proliferation, reducing the release of pro-inflammatory cytokines, and suppressing pro-inflammatory activation of macrophages (Figure 5). These results further verified the systematic inflammation caused by *E. faecium*, and such anti-inflammation treatment can improve the outcome. While the beneficial effects of hypothermia are evident, future research needs to explore whether hypothermia that commences post the onset of the disease can improve prognosis.

Mathematical models are widely used to study the host immune response and evaluate treatment effectiveness. Torres et al. developed a model that incorporates macrophage polarization to better understand the inflammatory response [21]. Ewald et al. derived a unique model emphasizing the role of alveolar epithelial cells in defending against invasive aspergillosis using dynamic optimization techniques [22]. Delattre et al. used a murine pneumonia model to record phage-bacteria interactions and characterized the synergy between phages and the host immune response using mathematical modelling [29]. Based on these studies, we developed a mathematical model to investigate the interactions between the host and bacteria, specifically focusing on the impact of hypothermia on the progression of *E. faecium* infection (Figure 6a-b). We examined the underlying mechanisms driving changes in both the origin and polarization of peritoneal macrophages following bacterial infection. Moreover, we found that hypothermia primarily reduces bacterial growth rates and decreases the rate of M1-polarization among recruited macrophages. The insights derived from our model significantly enhanced our understanding of immune responses in the context of *E. faecium* infection and provided a quantitative assessment of immunotherapy efficacy. Ultimately, this model may serve as a valuable guide for the development of more effective treatment strategies in the future.

The present study has several limitations. Firstly, despite the effectiveness of hypothermia in this murine model of septic peritonitis, it would be beneficial to explore the potential of combining hypothermia with other antibacterial treatments for further improving survival outcomes. Secondly, the inability to measure bacterial burden and immune cells *in vivo* poses a challenge in establishing a precise correlation between survival outcomes and these factors in individual animals, thereby impeding further personalized applications of the mathematical model. Future research should utilize *in vivo* detection techniques to develop personalized predictive models and deepen our understanding of host-bacteria interactions for new treatment targets. Lastly, we acknowledge that our findings are potentially limited by the modest sample size utilized in this study. While our biomathematical model offers valuable insights, we recognize the risk of underpowered statistical analysis and the possibility of spurious effects. To enhance the validity of our research, we will conduct a large study in the future to accurately develop a mathematical model that can further elaborate on the interaction between the host and pathogen.

## Conclusion

The present study provides important insights into the host-pathogen interactions in a murine model of septic peritonitis caused by VRE. The findings demonstrate that VRE infection leads to a significant activation of the cytokine-mediated signalling pathway. Furthermore, we showed that hypothermia can effectively improve survival outcomes by reducing bacterial burden and attenuating inflammation. Finally, mathematical model contributes to a better understanding of the mechanisms underlying VRE-associated septic peritonitis and further verified the effectiveness of hypothermia.

## Materials and methods

### Bacterial strain

A VRE strain, HJP554, was used in this study. The bacteria were inoculated in brain heart infusion (BHI) broth (BD Biosciences, Lawrence, KS) and grown overnight at 37°C in a shaking incubator at 220 rpm to stationary phase. Then the culture was diluted at 1:100 with BHI and maintained at 37°C for 3.5 h at 220 rpm. Next, the culture was further diluted and incubated for another 3 h to achieve OD_600_ 1.5. After that, the culture was adjusted to OD_600_ 1.5 for subsequent use, a concentration of approximately 3 × 10^8^ CFU/mL.

### Animals

BALB/c mice (females, 6–8 weeks, 16–18 g) were obtained from Vital River Laboratories (Beijing, China) for use in the experiments. Prior to the experiment, the mice were kept on a 12-hour light/dark cycle and adaptively raised for one week with free access to water and food. Animal breeding and experiments were approved by the Institute of Animal Care and Use Committee (IACUC-DWZX-2023-010) at the Academy of Military Medical Sciences. All animals were treated humanely in compliance with the National Institutes of Health guidelines for the use of experimental animals.

A total of 278 animals were used in this study. 140 animals were used in the experiments to determine sublethal dose, half-lethal dose, and lethal dose. In the experiments with sublethal dose, 10 animals were used for murine sepsis score and weight measurements, 28 animals were used for pathological examination and bacterial burden, 28 animals were used for RNA-seq analysis and cytokine detection, and 28 animals were used for flow cytometry. In the experiments with lethal dose, 20 animals were used for survival observation, 8 animals were used for bacterial burden and cytokine detection, and 16 animals were used for flow cytometry.

### Induction of septic peritonitis

Septic peritonitis was induced by intraperitoneal injection of 0.5 mL mixture containing *E. faecium*, 40% sterile rat faecal extract, and 10% sterile yeast extract. Sterile rat faecal extract was prepared by mixing crushed, dried rat faeces with 2 volumes of normal saline. The mixture was then autoclaved at 121°C and 15 lb of pressure for 20 min and subsequently centrifuged at 3000 rpm at 4°C for 10 min. The resulting supernatant (100% sterile rat faecal extract) was re-autoclaved under the same conditions. Sterile yeast extract was prepared by mixing yeast powder and normal saline at a weight ratio of 1:10. The mixture was also autoclaved and subsequently centrifuged under the same conditions as the sterile rat faecal extract. The resulting supernatant was re-autoclaved and designated as 100% sterile yeast extract.

### Induction of hypothermia

Twenty mice were randomly assigned to the hypothermia and normothermia groups (ten mice in each group). Hypothermia was initiated immediately after the inducing septic peritonitis with ice packs and an electric fan. Cooling was maintained at 32 ± 0.5°C for a duration of 2 h, and the target temperature was reached within 10 min. The mice in the normothermia group underwent the same procedure, but their target temperature was maintained at 37 ± 0.5°C. Following cooling, hypothermia animals were gradually rewarmed to 37°C and all animals were observed for 7 days.

### Murine sepsis score

We adopted the murine sepsis score to assess the severity of septic peritonitis [30]. The murine sepsis score assesses seven criteria, including appearance, level of consciousness, activity, response to stimulus, eyes, respiration rate, and respiration quality. Each criterion is scored between 0 and 4, and the total score reflects the clinical condition of mice with septic peritonitis.

### Collection of samples

Mice were anesthetized by an intraperitoneal injection of pentobarbital sodium (45 mg/kg). A peritoneal lavage was performed with 5 mL of sterile PBS using an 18-gauge needle. The PLF was collected in sterile polypropylene tubes (Plastipak; BD Biosciences). After collection of PLF, blood was drawn by cardiac puncture, with a sterile syringe, transferred to heparin-gel vacutainer tubes, and immediately placed on ice. Subsequently, the abdomen was opened and the liver, spleen, kidney, lung, and heart were harvested.

### Bacterial burden determination

The number of *E. faecium* CFU was determined in PLF, blood, liver, spleen, kidney, lung, and heart homogenates. To correct for the differences in organ weight, four times the weight (in milligrams) in microlitres of sterile saline was added. The organs were homogenized at 4°C with a tissue homogenizer (Biospec Products), which was carefully cleaned and disinfected with 70% ethanol after each homogenization. Next, serial 5-fold dilutions were made of each sample in sterile saline and 10 μL of each dilution was plated onto BHI plates containing 3.2% vancomycin (MedChemExpress, HY-B0671). The plates were incubated at 37°C under 5% CO_2_, and CFU were counted overnight, taking into account the dilution factor.

### Histological examination

The main organs, including the liver, spleen, kidney, lung, and heart, were dissected and subjected to haematoxylin-eosin staining (H&E) to examine for any morphological changes. The tissues were fixed in a general-purpose tissue fixation solution for at least 48 hours, embedded in paraffin, sectioned into 4 µm thick slices, and then stained with H&E. Photomicrographs were captured for analysis. The pathological score was evaluated by two independent pathologists. A semi-quantitative assessment was conducted by assessing various parameters, including inflammatory cell infiltration, necrosis, karyorrhexis, haemorrhage, atrophy, and presence of multinucleated giant cells. Each parameter was graded on a scale from 0 to 4, where 0 indicated normal, and higher numbers indicated increasing severity of injury. The total pathological score was calculated by summing the scores of all parameters.

### RNA extraction, library construction, sequencing and data preprocessing

Cell suspensions from the PLF were obtained after centrifugation for 5 min at 350 g and then the colleting cells were used for RNA extraction and transcriptome sequencing. For the extraction of total RNA, the PureLink™ RNA Mini Kit (Thermo Fisher Scientific) was utilized following the instructions provided by the manufacturer. To determine the purity of the extracted RNA, a NanoPhotometer (NanoDrop 2000c, USA) was used. The concentration and integrity of the RNA samples were assessed using an Agilent 2100 RNA nano 6000 assay kit (Agilent Technologies, CA, USA). All the samples were sent to Easyresearch Technology Company in China for sequencing. The cDNA libraries were sequenced using a paired-end strategy on the NovaSeq 6000 S4 platform, employing the NovaSeq 6000 S4 Reagent kit V1.5.

Sequencing data were filtered using a Perl script that: (1) removed reads containing the sequencing adapter; (2) removed reads with > 15% low-quality base ratio (base quality ≤ 5); and (3) removed reads whose unknown base (“N” base) ratio was > 5%. The resulting clean reads were stored in FASTQ format. Clean reads were mapped to the reference genome using HISAT2 (v2.1.0). Bowtie2 (v2.2.3) was used to align the clean reads to the reference coding gene set. We use Fastp for raw data quality control. The reads were mapped on the reference genome by STAR, followed by feature Counts to analyse the expression counts of each gene and then identify the Transcripts per million expressed genes.

### RNA-seq data analysis

RNA-seq data were analysed using R (R version 4.1.0). The EdgeR package was utilized to identify differentially expressed genes (DEGs) [31]. The selection criteria for identifying DEGs were an adjusted *p* value < 0.05 and a |log2 Fold Change| >1. To analyse the DEGs and gene sets, we employed the Bioconductor package clusterProfiler [32], which offers Gene Ontology and pathway-enrichment analysis using the Kyoto Encyclopedia of Genes and Genomes database. We also applied the Mfuzz R package to perform cluster analysis on the gene dynamic expression patterns, which was based on a fuzzy C-means algorithm with a cluster number of 4 and a fuzzy coefficient M of 2 [33].

### Cytokine and chemokine profiling

Serum and PLF were subjected to cytokine assays using the Cytokine and Chemokine Mouse Panel kit (eBioscience, CA, USA) on the Bio-Plex Multiplex Immunoassay System (Bio-Plex 200, CA, USA).

### Flow cytometry

PLF cell suspensions were incubated at room temperature for 10 min with zombie aqua viability dye (BioLegend), followed by a 10-min incubation on ice with blocking buffer containing 10% mouse serum with 0.25 µg/ml anti CD16/CD32 (BioLegend). The cells were then incubated with the indicated antibodies (Table S1) on ice for 30 min. Afterward, the cells were washed with FACS buffer (2 mM EDTA/0.5% BSA in PBS) and, if applicable, stained with streptavidin-conjugated or secondary antibodies. For intracellular staining, the cells were fixed/permeabilized using the Cytofix/Cytoperm (Bioscience) following the manufacturer’s protocol. The samples were acquired using FACS LSRFortessa (BD) and analysed using Flowjo (Version 10.4.1, Treestar). To analyse the data, doublets (based on Forward scatter area vs height) and dead cells (ZombieAqua positive) were excluded.

### Statistical analysis

The normality of data was tested by Kolmogorov-Smirnov test. Normal and non-normal distribution data were reported as mean ± standard deviation and medians with their interquartile ranges, respectively. One-way analysis of variance or repeated measures of two-way analysis of variance and post-hoc multiple comparison tests were used for normally distributed variables. Murine sepsis score was analysed non-parametrically using Kruskal-Wallis test and its post hoc comparisons. The Kaplan-Meier analysis and the log-rank test were used for survival analysis. These analysis were performed using GraphPad Prism version 8 (GraphPad Software). A P < 0.05 was considered statistically significant.

## Supplementary Material

Supplemental Material

## Data Availability

The data that support the findings of this study are openly available in Science Data Bank (https://www.scidb.cn/anonymous/UUpmcTJ5).
