# Supplementary material for: Unraveling host-pathogen dynamics in a murine Model of septic peritonitis induced by vancomycin-resistant Enterococcus faecium
Source: Virulence. 2024 Jul 1;15(1):2367659. doi: 10.1080/21505594.2024.2367659 (PMC11221476; doi:10.1080/21505594.2024.2367659)
Supplement: Supplemental Material [file KVIR_A_2367659_SM5771.zip › Supplementary information.pdf]

Supplementary information

Contents

Section S1. Model formulation 2
Table S1 Antibodies 7
Table S2. System variables in the mathematical model 8
Table S3. Model parameters and initial conditions 9
Figure S1. Bacterial burden and histological analysis of main organs after *E. faecium* infection 12
Figure S2. Number of DEGs and expression of four previously reported genes. 13
Figure S3. KEGG analysis revealed a comprehensive activation of cytokine-mediated signaling pathways 14
Figure S4. Cluster 3 and 4 of gene expression identified by Mfuzz cluster analysis and gene ontology analysis 15
Figure S5. Gating strategy 16
Figure S6. Simulated results of M0, M1, and M2 macrophages with different origins by mathematical model 18

Section S1. Model formulation

The model developed in this manuscript tracks the signaling and resulting immune response within the peritoneal cavity. We do not explicitly model the blood component and all variables represent local levels. To create this model, previous models of immune response to bacteria[1,2] have been adapted to include growth and killing of pathogen, the disappearance of large peritoneal macrophages (LPMs), polarization of macrophages among phenotypes M0, M1 and M2, interactions among macrophages of different origins, release of cytokines, as well as recruitment of neutrophils.

System variables include cell populations given by M_0 (M0 macrophages), M_1 (M1 macrophages), M_2 (M2 macrophages), L (LPMs) and N (neutrophils), E (*E. faecium*), C_1 (cytokine 1), C_2 (cytokine 2). M_0, M_1 and M_2 can be further classified into resident, recruited, and monocyte-derived subpopulations, with following relationships: $M_0 = M_0^{rs} + M_0^{rc} + M_0^{m}$, $M_1 = M_1^{rs} + M_1^{rc} + M_1^{m}$, $M_2 = M_2^{rs} + M_2^{rc} + M_2^{m}$ (see Table S2 for details). Model parameters and estimates for growth rates, activation, transition, depletion, and interactions are specified in Table S3. The healthy peritoneal cavity is impermeable and is assumed to be almost sterile before inflammatory stimulation, without any pathogens. According to our above results (Figure 4), the resident cells only include L, M_0^{rs}, M_1^{rs} and M_2^{rs}.

When bacteria are injected into the peritoneal cavity, the number of bacteria is influenced by growth, depletion, killing of immune cells. We assumed that they growth exponentially in the absence of immune cells and the growth rate is affected by the body temperature. We modeled killing of E by $L + M_0$, M_1 and N, and assumed the killing rate is highest for N, followed by M_1 and $L + M_0$ ($p_{36} < p_{37} < p_{38}$), based on the experimental results (Figure S5b). We obtained the following description of bacteria dynamics:

$$\frac{dE}{dt} = \underbrace{p_{35}E}_{\text{growth}} - \underbrace{p_{36}f(E,p_{45})(L + M_0)}_{\text{killing by } L+M_0} - \underbrace{p_{37}f(E,p_{45})M_1}_{\text{killing by } M_1} - \underbrace{p_{38}f(E,p_{45})N}_{\text{killing by } N} \qquad \text{(S1)}$$

where $f(E,p_{45}) = \frac{E}{1+p_{45}E}$, $f(\cdot)$ is the hill function, provides fit for different doses of bacteria.

Subsequent to bacterial infection, macrophages form the first line of cellular defense, but they are not capable of effective elimination of the bacteria after phagocytosis. LPMs (L) can recruit monocyte-derived cells and neutrophils by adhering to the mesothelium, forming multilayered cellular aggregates, and releasing of cytokines (C) [3].

$$\frac{dL}{dt} = -\underbrace{p_5 f(E, p_{45}) L}_{\text{lysis by bacteria}} \quad \text{(S2)}$$

In addition to LPMs, F4/80^{Med} macrophages also play an important role in response to *E. faecium*. Macrophages can be classified into three types based on their origins: resident, recruited, and monocyte-derived cells (Figure 6b). There exists a conversion relationship between recruited and monocyte-derived cells, where monocyte-derived macrophages gradually transformed into recruited macrophages as the expression of Ly6C decreases over time (Figure S6c). Furthermore, macrophages can differentiate into three subsets, M0, M1, and M2, each exhibiting distinct biological features in terms of receptor expression, oxidative and nonoxidative antimicrobial defenses, cytokine production, and antigen presentation [4]. M1 macrophages, often referred to as classically activated macrophages, have been extensively studied in response to bacterial infection and are characterized by elevated expression of MHC-II, CD80, and CD86 costimulatory molecules [4]. On the other hand, M2 macrophages, also known as alternatively activated macrophages, produce anti-inflammatory cytokines and tend to dominate the response, suppressing the inflammatory and Th1 adaptive immune response while promoting a Th2 response. M0 macrophages, which do not exhibit the characteristics of either M1 or M2 macrophages, are considered resting or non-activated macrophages.

We assumed that there is a state conversion relationship among M0, M1, and M2 macrophages. Under bacterial stimulation, M0 can transform into either M1 or M2. However, M1 and M2 cannot convert back to M0. It is important to note that all macrophages can only be recruited to M_0^m and M_0^{rc}, meaning that recruited cells cannot directly transform into M1 or M2 and cannot be recruited into M_0^{rs}. We also

assumed that the state conversion of M_0^{rc} to M_1^{rc} is affected by body temperature, as shown in Figure 5e and Figure S6f.

$$\frac{dM_0^{rs}}{dt} = -\underbrace{p_6 f(E,p_{45})M_0^{rs}}_{\text{transfer to } M_1^{rs}} - \underbrace{p_7 M_0^{rs}}_{\text{depletion}} - \underbrace{p_8 f(E,p_{45})M_0^{rs}}_{\text{transfer to } M_2^{rs}} - \underbrace{p_9 f(E,p_{46})M_0^{rs}}_{\text{lysis by bacteria}} \tag{S3}$$

$$\begin{aligned}\frac{dM_0^{m}}{dt} = &\underbrace{\frac{p_{10}C_1}{1+p_{11}C_1}}_{\text{recruitment}} - \underbrace{p_{12}M_0^{m}}_{\text{depletion}} - \underbrace{p_{13}M_0^{m}}_{\text{transfer to } M_0^{rc}} - \underbrace{(p_{14}f(E,p_{45}) + p_{15}M_1)M_0^{m}}_{\text{transfer to } M_1^{m}} \\ &- \underbrace{p_{16}f(E,p_{45})M_0^{m}}_{\text{transfer to } M_2^{m}} - \underbrace{p_{17}f(E,p_{45})M_0^{m}}_{\text{lysis by bacteria}}\end{aligned} \tag{S4}$$

$$\begin{aligned}\frac{dM_0^{rc}}{dt} = &\underbrace{p_{18} + \frac{p_{19}C_1}{1+p_{20}C_1}}_{\text{recruitment}} - \underbrace{p_{21}M_0^{rc}}_{\text{depletion}} + \underbrace{p_{13}M_0^{m}}_{\text{transfer from } M_0^{m}} - \underbrace{p_{22}f(E,p_{46})M_0^{rc}}_{\text{transfer to } M_1^{rc}} \\ &- \underbrace{p_{23}f(E,p_{45})M_0^{rc}}_{\text{transfer to } M_2^{rc}} - \underbrace{p_{24}f(E,p_{45})M_0^{rc}}_{\text{lysis by bacteria}}\end{aligned} \tag{S5}$$

$$\frac{dM_1^{rs}}{dt} = \underbrace{p_6 f(E,p_{45})M_0^{rs}}_{\text{transfer from } M_0^{rs}} - \underbrace{p_{25}M_1^{rs}}_{\text{depletion}} - \underbrace{p_{26}f(E,p_{45})M_1^{rs}}_{\text{lysis by bacteria}} \tag{S6}$$

$$\frac{dM_1^{m}}{dt} = \underbrace{(p_{14}f(E,p_{45}) + p_{15}M_1)M_0^{m}}_{\text{transfer from } M_0^{m}} - \underbrace{p_{27}M_1^{m}}_{\text{depletion}} - \underbrace{p_{28}M_1^{m}}_{\text{transfer to } M_1^{rc}} - \underbrace{p_{29}f(E,p_{45})M_1^{m}}_{\text{lysis by bacteria}} \tag{S7}$$

$$\frac{dM_1^{rc}}{dt} = \underbrace{p_{22}f(E,p_{46})M_0^{rc}}_{\text{transfer to } M_1^{rc}} - \underbrace{p_{30}M_1^{rc}}_{\text{depletion}} + \underbrace{p_{28}M_1^{m}}_{\text{transfer from } M_1^{m}} - \underbrace{p_{31}f(E,p_{45})M_1^{rc}}_{\text{lysis by bacteria}} \tag{S8}$$

$$\frac{dM_2^{rs}}{dt} = \underbrace{p_8 f(E,p_{45})M_0^{rs}}_{\text{transfer from } M_0^{rs}} - \underbrace{p_{32}M_2^{rs}}_{\text{depletion}} \tag{S9}$$

$$\frac{dM_2^{m}}{dt} = \underbrace{p_{16} f(E,p_{45})M_0^{m}}_{\text{transfer from } M_0^{m}} - \underbrace{p_{33}M_2^{m}}_{\text{depletion}} \tag{S10}$$

$$\frac{dM_2^{rc}}{dt} = \underbrace{p_{23} f(E,p_{45})M_0^{rc}}_{\text{transfer from } M_0^{rc}} - \underbrace{p_{34}M_2^{rc}}_{\text{depletion}} \tag{S11}$$

An important role of macrophages is the release of pro-inflammatory cytokines in response to *E. faecium*, and mediating the recruitment of monocyte-derived macrophage, recruited macrophages, and neutrophils. Thus, we model the dynamics of two pro-inflammatory cytokines (C_1 and C_2), C_1 for macrophages recruitment and C_2 for neutrophils recruitment:

$$\frac{dC_1}{dt} = \underbrace{p_{39}f(E,p_{47})(L+M_0)}_{\text{inflammation by } L+M_0} + \underbrace{p_{40}f(E,p_{47})M_1}_{\text{inflammation by } M_1} - \underbrace{p_{41}C_1}_{\text{decay}} \tag{S12}$$

$$\frac{dC_2}{dt} = \underbrace{p_{42}f(E,p_{47})(L+M_0)}_{\text{inflammation by } L+M_0} + \underbrace{p_{43}f(E,p_{47})M_1}_{\text{inflammation by } M_1} - \underbrace{p_{44}C_2}_{\text{decay}} \tag{S13}$$

Although neutrophils don't reside in peritoneal cavity, they are recruited in large quantities from the blood in response to *E. faecium* infection by pro-inflammatory cytokines like IL-6, IL-12p70 and CXCL2. The kinetics of a neutrophil population (N) is described by:

$$\frac{dN}{dt} = \underbrace{\frac{p_1 C_2}{1 + p_2 C_2}}_{\text{recruitment}} - \underbrace{p_3 N}_{\text{depletion}} - \underbrace{p_4 NE}_{\text{lysis by bacteria}} \qquad \text{(S14)}$$

The model given by Eqs. S1-S14 was fit to experimental data using an iterative method called the trust-region algorithm [5], for parameter estimation. We assumed all parameters are non-negative. The fitting procedure was then performed iteratively via weighted least squares with merit function [1]

$$\chi^2(\mathbf{p}) = \sum_{i=1}^{n} \sum_{j=1}^{m} \left(\frac{y_{i,j} - \hat{y}_{i,j}(\mathbf{p})}{\sigma_i} \right)^2 \qquad \text{(S15)}$$

with $\mathbf{p}$ the vector of estimated parameters, $y_{i,j}$ the observations, $\hat{y}_{i,j}(\mathbf{p})$ given the parameter estimates, σ_i the standard errors of $y_{i,\cdot}$, and n equal to the total number of system variables, m equal to the total number of time points. Then the robust adaptive Metropolis (RAM) algorithm [6] was used to conduct the Markov Chain Monte Carlo (MCMC) procedure and estimate the target distribution of $\mathbf{p}$. The algorithm ran for 3×10^6 iterations after a burn-in of 10^6 iterations, with the Geweke convergence diagnostic method employed to assess the convergence. Finally, we reliably obtained the distribution and 95% confidence interval of $\mathbf{p}$ (Table S3).

References

1. Torres, M., Wang, J., Yannie, P.J., Ghosh, S., Segal, R.A., and Reynolds, A.M. (2019). Identifying important parameters in the inflammatory process with a mathematical model of immune cell influx and macrophage polarization. PLoS Comput. Biol. *15*, 1–27. 10.1371/journal.pcbi.1007172.
2. Ewald, J., Rivieccio, F., Radosa, L., Schuster, S., Brakhage, A.A., and Kaleta, C. (2021). Dynamic optimization reveals alveolar epithelial cells as key mediators of host defense in invasive aspergillosis. PLoS Comput. Biol. *17*, 1–21. 10.1371/journal.pcbi.1009645.
3. Vega-Pérez, A., Villarrubia, L.H., Godio, C., Gutiérrez-González, A., Feo-Lucas, L., Ferriz, M., Martínez-Puente, N., Alcaín, J., Mora, A., Sabio, G., et al. (2021). Resident macrophage-dependent immune cell scaffolds drive anti-bacterial defense in the peritoneal cavity. Immunity *54*, 2578-2594.e5. 10.1016/j.immuni.2021.10.007.
4. Day, J., Friedman, A., and Schlesinger, L.S. (2009). Modeling the immune rheostat of macrophages in the lung in response to infection. Proc. Natl. Acad. Sci. U. S. A. *106*, 11246–11251. 10.1073/pnas.0904846106.

5. Coleman, T.F., and Li, Y. (1996). An interior trust region approach for nonlinear minimization subject to bounds. SIAM J. Optim. *6*, 418–445. 10.1137/0806023.
6. Vihola, M. (2012). Robust adaptive Metropolis algorithm with coerced acceptance rate. Stat. Comput. *22*, 997–1008. 10.1007/s11222-011-9269-5.

Table S1. Antibodies

Antibody	Clone	Source	Fluorochrome	Catalogue#	Dilution
Purified Rat Anti-Mouse CD16/CD32 (Mouse BD Fc Block™)	2.4G2	Bioscience		5531542	1:50
CD11b	M1/70	Bioscience	FITC	557396	1:125
F4/80	BM8	ebioscience	APC	17-4801-82	1:100
CD11c	N418	Biolegend	BV510	117337	1:100
Ly6C	HK1.4	Biolegend	Percp-Cy5.5	128011	1:100
Ly6G	1A8	Biolegend	BV650	127641	1:100
CD11b	M1/70	Biolegend	BV605	101237	1:100
MHC-II	2G9	Bioscience	BUV395	743876	1:100
CD86	GL-1	Biolegend	PE-Cy7	105014	1:100
CD206	MMR	Biolegend	BV421	141717	1:100
CD102	3C4(MIC2/4)	Biolegend	AF647	105612	1:100
Zombie NIR™ Fixable Viability kit		Biolegend	APC-Cy7	423105	1:1000

Table S2. System variables in the mathematical model

Variable	Subpopulation	Name	Cell marker
N	-	Neutrophil	Ly6G^{+}
L	-	Large peritoneal macrophage	F4/80^{Hi} PKH26-PCL^{Hi}
M_0	M_0^{rs}	Resident M0 macrophages	F4/80^{Med} PKH26-PCL^{Hi}
	M_0^{m}	Monocyte-derived M0 macrophages	F4/80^{Med} PKH26-PCL^{Lo} Ly6C^{+}
	M_0^{rc}	Recruited M0 macrophages	F4/80^{Med} PKH26-PCL^{Lo} Ly6C^{-}
M_1	M_1^{rs}	Resident M1 macrophages	F4/80^{Med} PKH26-PCL^{Hi} CD86^{+}
	M_1^{m}	Monocyte-derived M1 macrophages	F4/80^{Med} PKH26-PCL^{Lo} Ly6C^{+} CD86^{+}
	M_1^{rc}	Recruited M1 macrophages	F4/80^{Med} PKH26-PCL^{Lo} Ly6C^{-} CD86^{+}
M_2	M_2^{rs}	Resident M2 macrophages	F4/80^{Med} PKH26-PCL^{Hi} CD206^{+}
	M_2^{m}	Monocyte-derived M2 macrophages	F4/80^{Med} PKH26-PCL^{Lo} Ly6C^{+} CD206^{+}
	M_2^{rc}	Recruited M2 macrophages	F4/80^{Med} PKH26-PCL^{Lo} Ly6C^{-} CD206^{+}
C_1	-	Cytokine levels	-
C_2	-	Cytokine levels	-
E	-	*E. faecium*	-

Table S3. Model parameters and initial conditions

Parameter	Description	Mean	95%CI	Source
p_1	Recruited rate of neutrophil by C_2	2.71	(6.51×10^{-1}, 6.28)	MCMC
p_2	Damping rate of neutrophil recruitment	7.14×10^{-1}	(1.35×10^{-1}, 1.76)	MCMC
p_3	Rate of neutrophil depletion	4.88×10^{-1}	(2.79×10^{-1}, 7.96×10^{-1})	MCMC
p_4	Lysis rate of neutrophil by *E. faecium*	2.91	(1.03, 6.04)	MCMC
p_5	Lysis rate of LPM by *E. faecium*	3.66	(2.73, 4.67)	MCMC
p_6	Transfer rate from M_0^{rs} to M_1^{rs}	7.37×10^{-3}	(2.48×10^{-4}, 2.33×10^{-2})	MCMC
p_7	Rate of M_0^{rs} depletion	4.30×10^{-2}	(4.73×10^{-4}, 2.10×10^{-1})	MCMC
p_8	Transfer rate from M_0^{rs} to M_2^{rs}	8.06×10^{-2}	(2.40×10^{-3}, 2.73×10^{-1})	MCMC
p_9	Lysis rate of M_0^{rs} by *E. faecium*	3.36	(1.07, 4.80)	MCMC
p_{10}	Recruited rate of M_0^{m}	5.04×10^{-1}	(2.53×10^{-1}, 7.87×10^{-1})	MCMC
p_{11}	Damping rate of M_0^{m} recruitment	1.72	(7.66×10^{-1}, 2.91)	MCMC
p_{12}	Rate of M_0^{m} depletion	1.28×10^{-2}	(1.81×10^{-4}, 5.23×10^{-2})	MCMC
p_{13}	Transfer rate from M_0^{m} to M_0^{rc}	1.60×10^{-2}	(1.94×10^{-4}, 6.23×10^{-2})	MCMC
p_{14}	Transfer rate from M_0^{m} to M_1^{m} by *E. faecium*	5.78	(3.98, 7.96)	MCMC
p_{15}	Transfer rate from M_0^{m} to M_1^{m} by M_1	2.99×10^{-2}	(1.09×10^{-3}, 5.85×10^{-2})	MCMC
p_{16}	Transfer rate from M_0^{m} to M_2^{m}	6.91×10^{-1}	(4.94×10^{-1}, 9.33×10^{-1})	MCMC
p_{17}	Lysis rate of M_0^{m} by *E. faecium*	4.94×10^{-1}	(1.42×10^{-2}, 1.71)	MCMC
p_{18}	Recruited rate of M_0^{rc}	1.45×10^{-1}	(6.90×10^{-2}, 2.51×10^{-1})	MCMC
p_{19}	Recruited rate of M_0^{rc} by C_1	5.84	(2.69, 1.08×10^{1})	MCMC
p_{20}	Damping rate of M_0^{rc} recruitment	1.50	(7.52×10^{-1}, 2.44)	MCMC
p_{21}	Rate of M_0^{rc} depletion	1.59×10^{-1}	(9.97×10^{-2} 2.42×10^{-1})	MCMC
p_{22}	Transfer rate from M_0^{rc} to M_1^{rc} for normothermia	1.68×10^{-2}	(7.94×10^{-3}, 3.11×10^{-2})	MCMC
	Transfer rate from M_0^{rc} to M_1^{rc} for hypothermia	1.19×10^{-2}	(5.80×10^{-3}, 2.28×10^{-2})	MCMC
p_{23}	Transfer rate from M_0^{rc} to M_2^{rc}	1.39×10^{-1}	(1.04×10^{-1}, 1.79×10^{-1})	MCMC
p_{24}	Lysis rate of M_0^{rc} by *E. faecium*	7.46	(2.88, 1.53×10^{1})	MCMC

p_{25}	Rate of M_1^{rs} depletion	1.59×10^{-2}	(1.01×10^{-2}, 2.49×10^{-2})	MCMC
p_{26}	Lysis rate of M_1^{rs} by *E. faecium*	5.46×10^{-1}	(3.19×10^{-1}, 8.48×10^{-1})	MCMC
p_{27}	Rate of M_1^{m} depletion	3.30×10^{-2}	(1.98×10^{-3}, 8.05×10^{-2})	MCMC
p_{28}	Transfer rate from M_1^{m} to M_1^{rc}	6.05×10^{-2}	(3.05×10^{-2}, 9.23×10^{-2})	MCMC
p_{29}	Lysis rate of M_1^{m} by *E. faecium*	1.67×10^{-1}	(5.17×10^{-3}, 5.85×10^{-1})	MCMC
p_{30}	Rate of M_1^{rc} depletion	5.25×10^{-2}	(2.99×10^{-2}, 7.79×10^{-2})	MCMC
p_{31}	Lysis rate of M_1^{rc} by *E. faecium*	1.67	(1.17×10^{-1}, 4.28)	MCMC
p_{32}	Rate of M_2^{rs} depletion	1.84×10^{-1}	(1.53×10^{-1}, 2.26×10^{-1})	MCMC
p_{33}	Rate of M_2^{m} depletion	1.12×10^{-1}	(7.88×10^{-2}, 1.56×10^{-1})	MCMC
p_{34}	Rate of M_2^{rc} depletion	1.76×10^{-2}	(1.34×10^{-2}, 2.28×10^{-2})	MCMC
p_{35}	Growth rate of *E. faecium* for normothermia	1.83×10^{-1}	(1.63×10^{-1}, 2.01×10^{-1})	MCMC
	Growth rate of *E. faecium* for hypothermia	4.00×10^{-2}	(3.55×10^{-2}, 4.51×10^{-2})	MCMC
p_{36}	Killing rate of *E. faecium* by LPM and M_0	3.64×10^{-1}	(6.34×10^{-2}, 6.01×10^{-1})	MCMC
p_{37}	Killing rate of *E. faecium* by M_1	5.75×10^{-1}	(6.05×10^{-1}, 9.58×10^{-1})	MCMC
p_{38}	Killing rate of *E. faecium* by neutrophil	7.65×10^{-1}	(4.76×10^{-3}, 6.35×10^{-1})	MCMC
p_{39}	Release rate of C_1 by LPM and M_0	2.01×10^{-1}	(8.81×10^{-3}, 5.86×10^{-1})	MCMC
p_{40}	Release rate of C_1 by M_1	4.48	(1.36, 8.44)	MCMC
p_{41}	Decay rate of C_1	1.79	(1.19, 2.20)	MCMC
p_{42}	Release rate of C_2 by LPM and M_0	3.48×10^{-1}	(9.79×10^{-3}, 1.22)	MCMC
p_{43}	Release rate of C_2 by M_1	4.86	(5.11×10^{-1}, 1.18×10^{1})	MCMC
p_{44}	Decay rate of C_2	7.14×10^{-2}	(5.14×10^{-2}, 9.39×10^{-2})	MCMC
p_{45}	Inhibition rate of *E. faecium* in host-pathogen interplay	1.36×10^{1}	(1.03×10^{1}, 1.73×10^{1})	MCMC
p_{46}	Inhibition rate of *E. faecium* in M_1^{rc} activation	9.17×10^{-2}	(3.43×10^{-2}, 1.57×10^{-1})	MCMC
p_{47}	Inhibition rate of *E. faecium* in cytokine release	5.40×10^{-1}	(1.61×10^{-1}, 1.05)	MCMC
$N(0)$	Initial value of neutrophils	4.52×10^{4}	-	Measured
$L(0)$	Initial value of LPM	1.2723×10^{4}	-	Measured
$M_0^{rs}(0)$	Initial value of M_0^{rs}	5.77×10^{3}	-	Measured

$M_0^m(0)$	Initial value of M_0^m	2.31×10^3	-	Measured
$M_0^{rc}(0)$	Initial value of M_0^{rc}	5.42×10^4	-	Measured
$M_1^{rs}(0)$	Initial value of M_1^{rs}	277	-	Measured
$M_1^m(0)$	Initial value of M_1^m	4.69×10^3	-	Measured
$M_1^{rc}(0)$	Initial value of M_1^{rc}	2.96×10^3	-	Measured
$M_2^{rs}(0)$	Initial value of M_2^{rs}	2.26×10^3	-	Measured
$M_2^m(0)$	Initial value of M_2^m	38	-	Measured
$M_2^{rc}(0)$	Initial value of M_2^{rc}	2.06×10^3	-	Measured
$E(0)$	Initial value of *E. faecium* of sublethal dose	9.03×10^7	-	Measured
	Initial value of *E. faecium* of lethal dose	1.99×10^9	-	Measured
$C_1(0)$	Initial value of cytokine 1	0	-	Assumed
$C_2(0)$	Initial value of cytokine 2	0	-	Assumed

CI: confidence interval. MCMC: Markov Chain Monte Carlo.

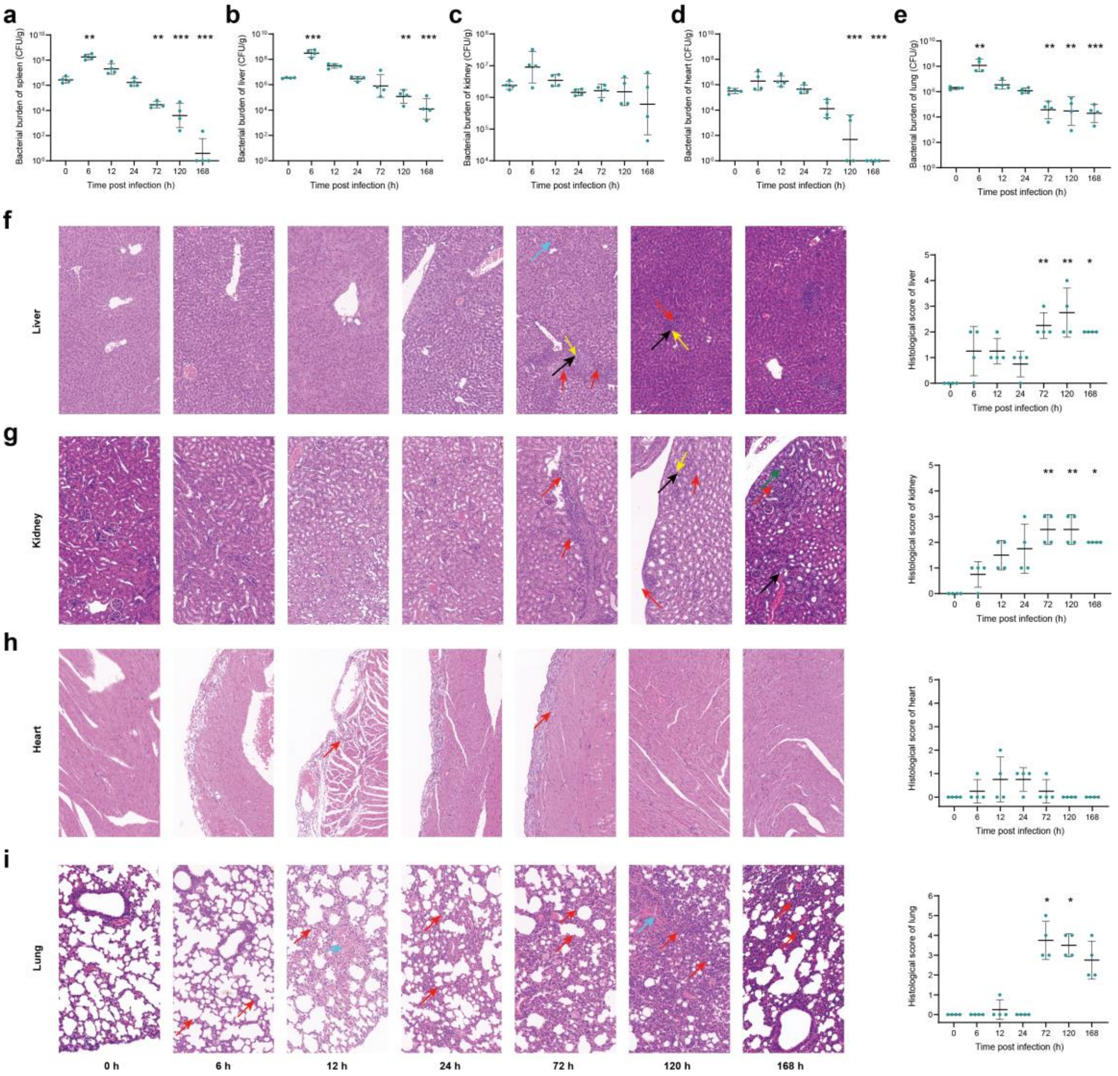

Figure S1. Bacterial burden and histological analysis of main organs after *E. faecium* infection. Bacterial burden in spleen (a), liver (b), kidney (c), heart (d), and lung (e). Histological analysis in liver (f), kidney (g), heart (h), and lung (i). Red arrows indicate inflammatory cell infiltration, light blue arrows indicate hemorrhage, yellow arrows indicate karyorrhexis, orange arrows indicate the presence of multinucleated giant cells, green arrows indicate atrophy, dark arrows indicate necrosis. (original magnification ×20). *: $P<0.05$, **: $P<0.01$, ***: $P<0.001$ when compared to 0 h.

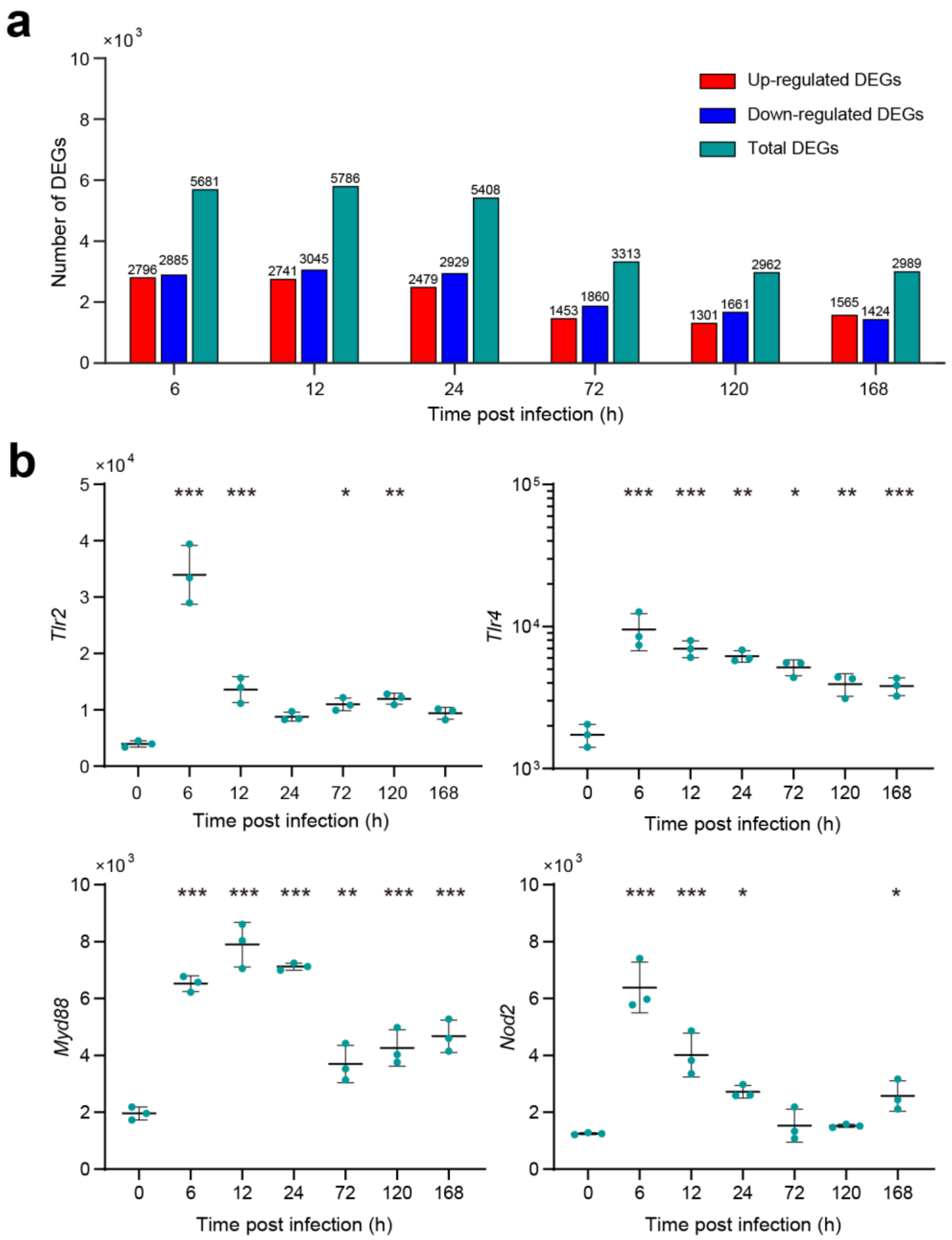

Figure S2. Number of DEGs and expression of four previously reported genes. (a) Number of DEGs. (b) Reads of *Tlr2*, *Tlr4*, *Myd88* and *Nod2*. DEGs: differentially expressed genes. *: P<0.05, **: P<0.01, ***: P<0.001 when compared to 0 h.

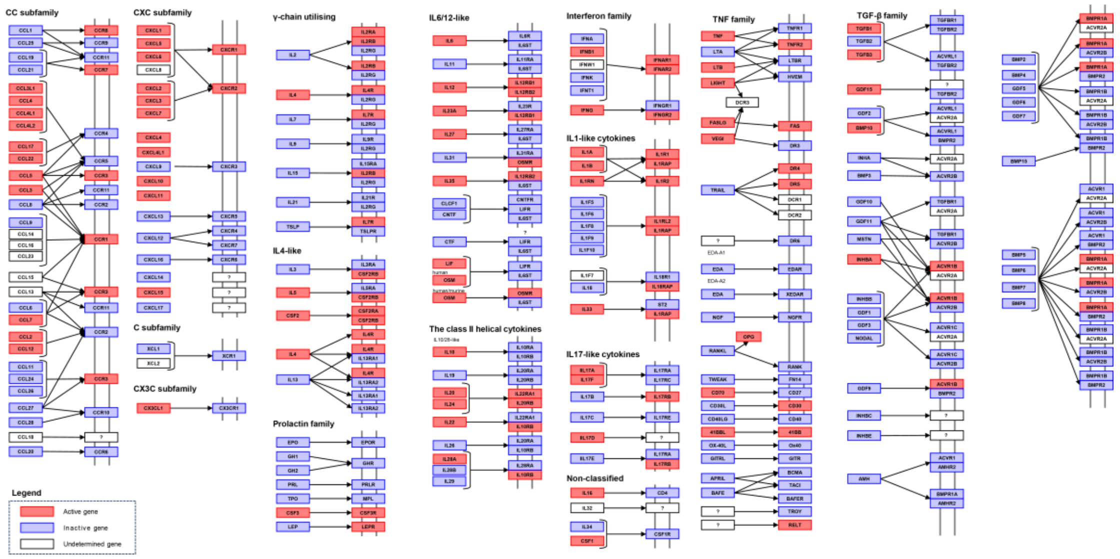

Figure S3. KEGG analysis revealed a comprehensive activation of cytokine-mediated signaling pathways.

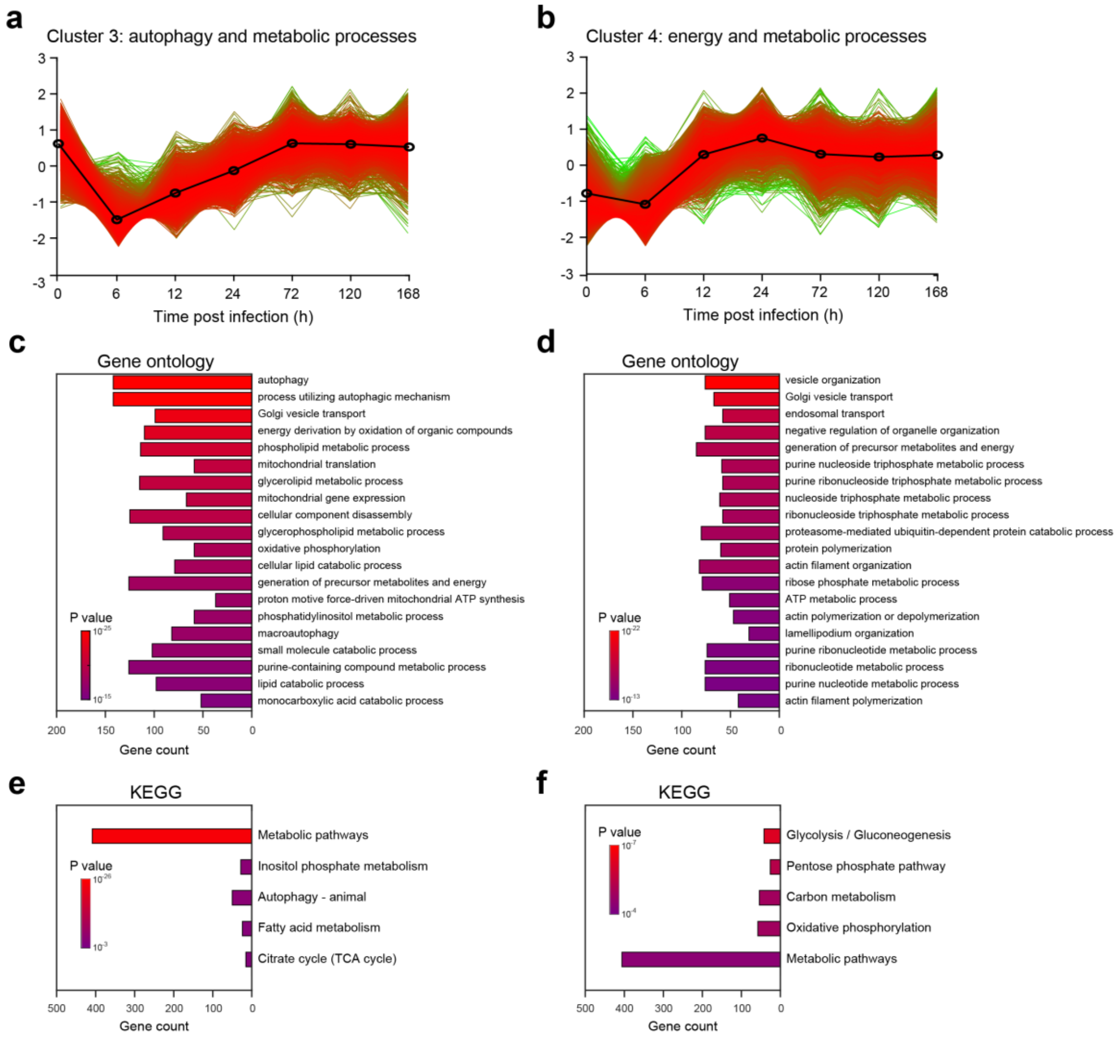

Figure S4. Cluster 3 and 4 of gene expression identified by Mfuzz cluster analysis and gene ontology analysis. (a-b) Cluster 3 and 4 of gene expression. (c-d) Gene ontology analysis of cluster 3 and 4. (e-f) KEGG enrichment analysis of cluster 3 and 4.

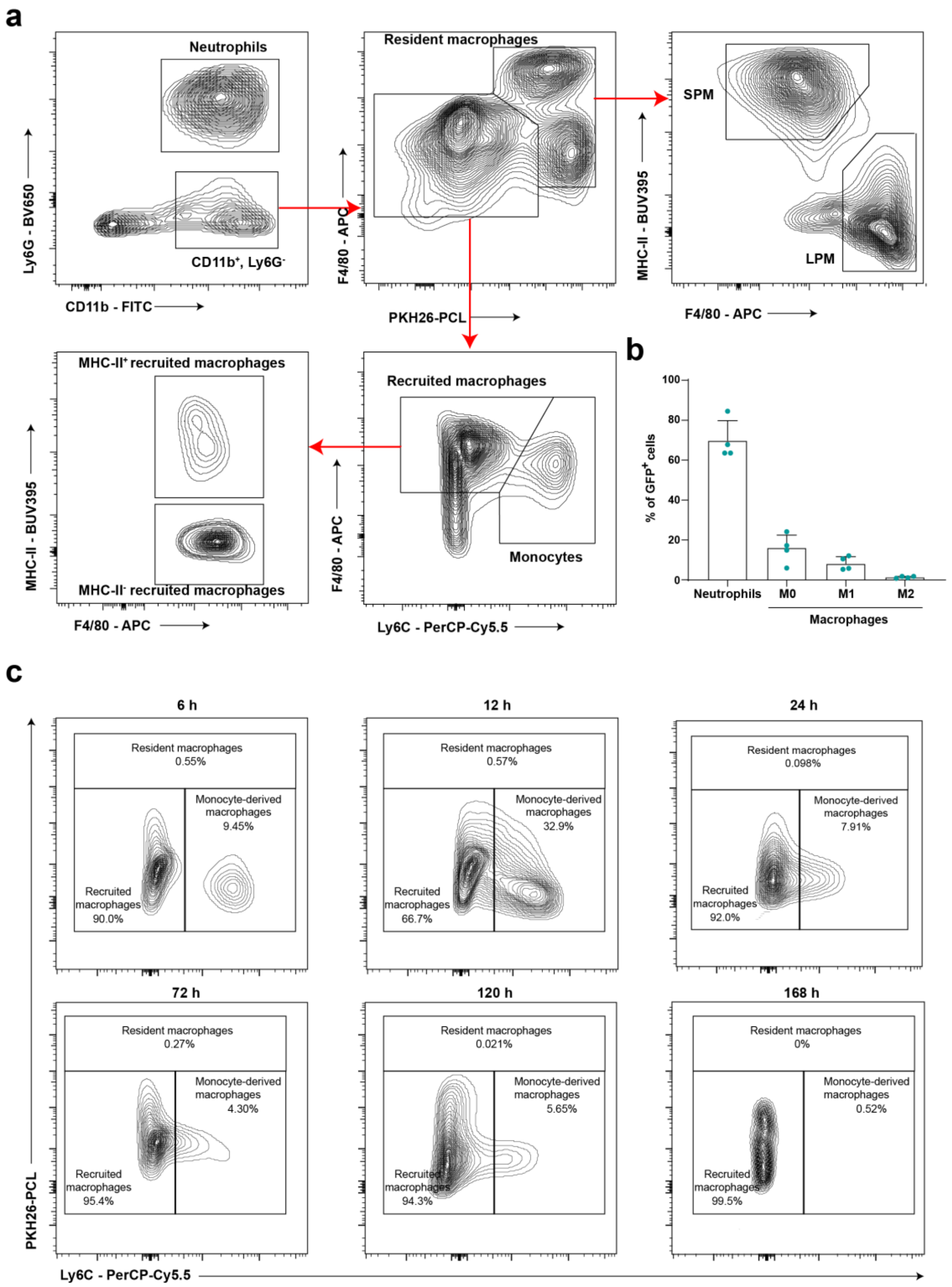

Figure S5. Gating strategy. (a) Gating strategy for flow cytometry analysis. (b) Percentage of GFP$^+$ cells. Dead GFP$^+$ *E. coli* was intraperitoneally injected at 12 h after *E. faecium* infection. Then peritoneal fluid was used for flow cytometry analysis. Despite the higher proportion of GFP$^+$ M0 cells compared to GFP$^+$ M1 cells, there is a significantly larger number of M0 cells than M1 cells. As a result, M1 exhibits a stronger phagocytic ability in comparison to M0. (c) Ly6C expression of

monocyte-derived macrophages decreased overtime. LPM, large peritoneal macrophages. SPM, small peritoneal macrophages.

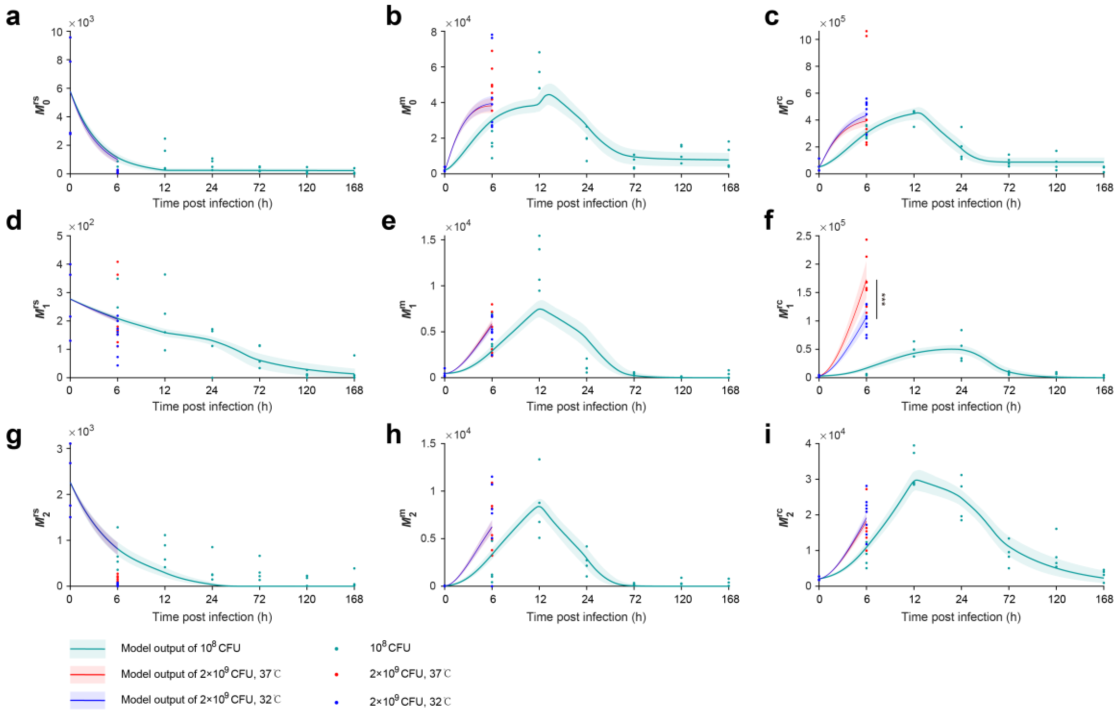

Figure S6. Simulated results of M0, M1, and M2 macrophages with different origins by mathematical model. (a) M_0^{rs}, (b) M_0^{m}, (c) M_0^{rc}, (d) M_1^{rs}, (e) M_1^{m}, (f) M_1^{rc}, (g) M_2^{rs}, (h) M_2^{m}, (i) M_2^{rc}. ***: P<0.001.
