# Supplementary figures and images for: Unraveling host-pathogen dynamics in a murine Model of septic peritonitis induced by vancomycin-resistant Enterococcus faecium
Source: Virulence. 2024 Jul 1;15(1):2367659. doi: 10.1080/21505594.2024.2367659 (PMC11221476; doi:10.1080/21505594.2024.2367659)

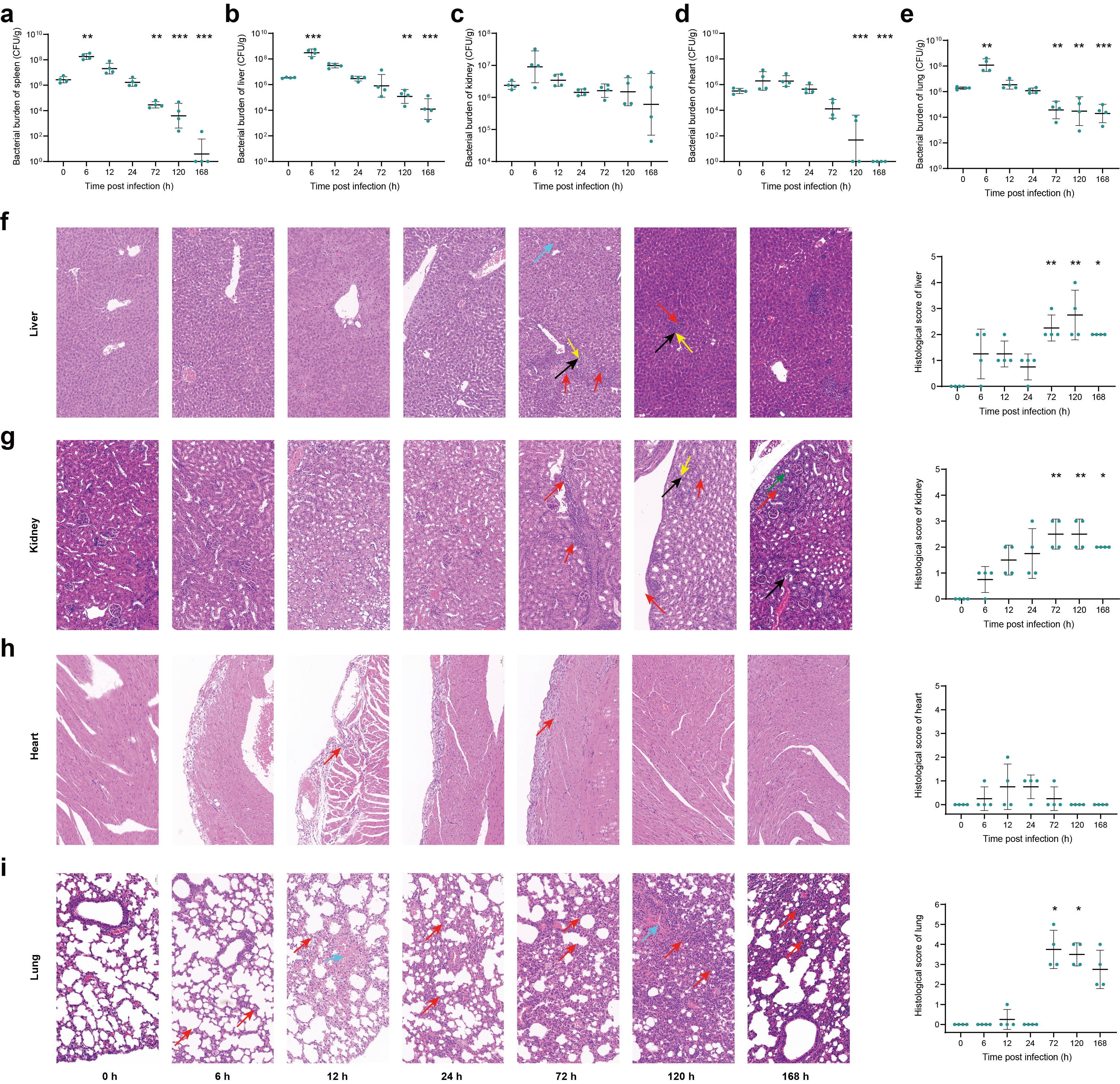

Supplement: Supplemental Material [file KVIR_A_2367659_SM5771.zip › Figure S1.png]

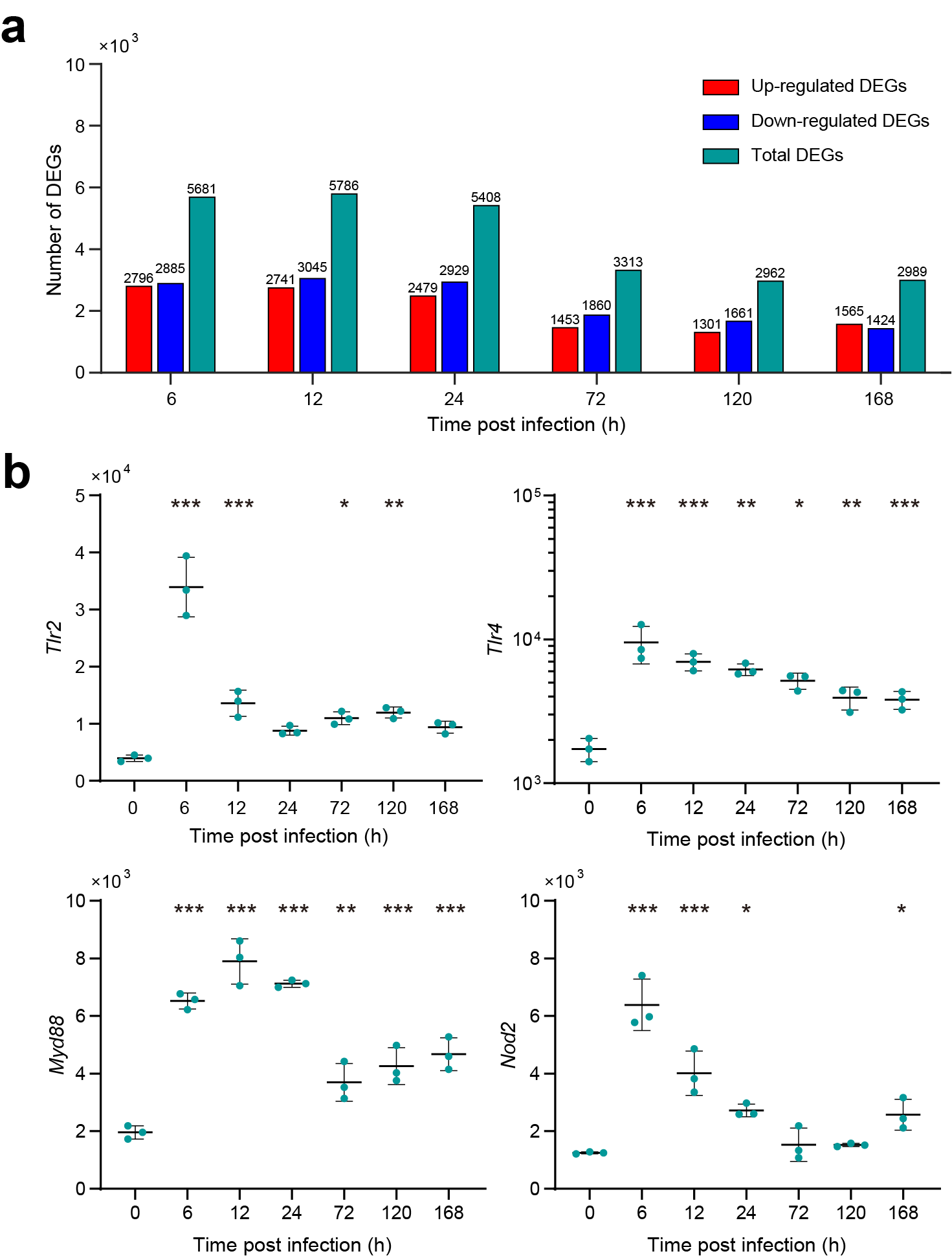

Supplement: Supplemental Material [file KVIR_A_2367659_SM5771.zip › Figure S2.png]

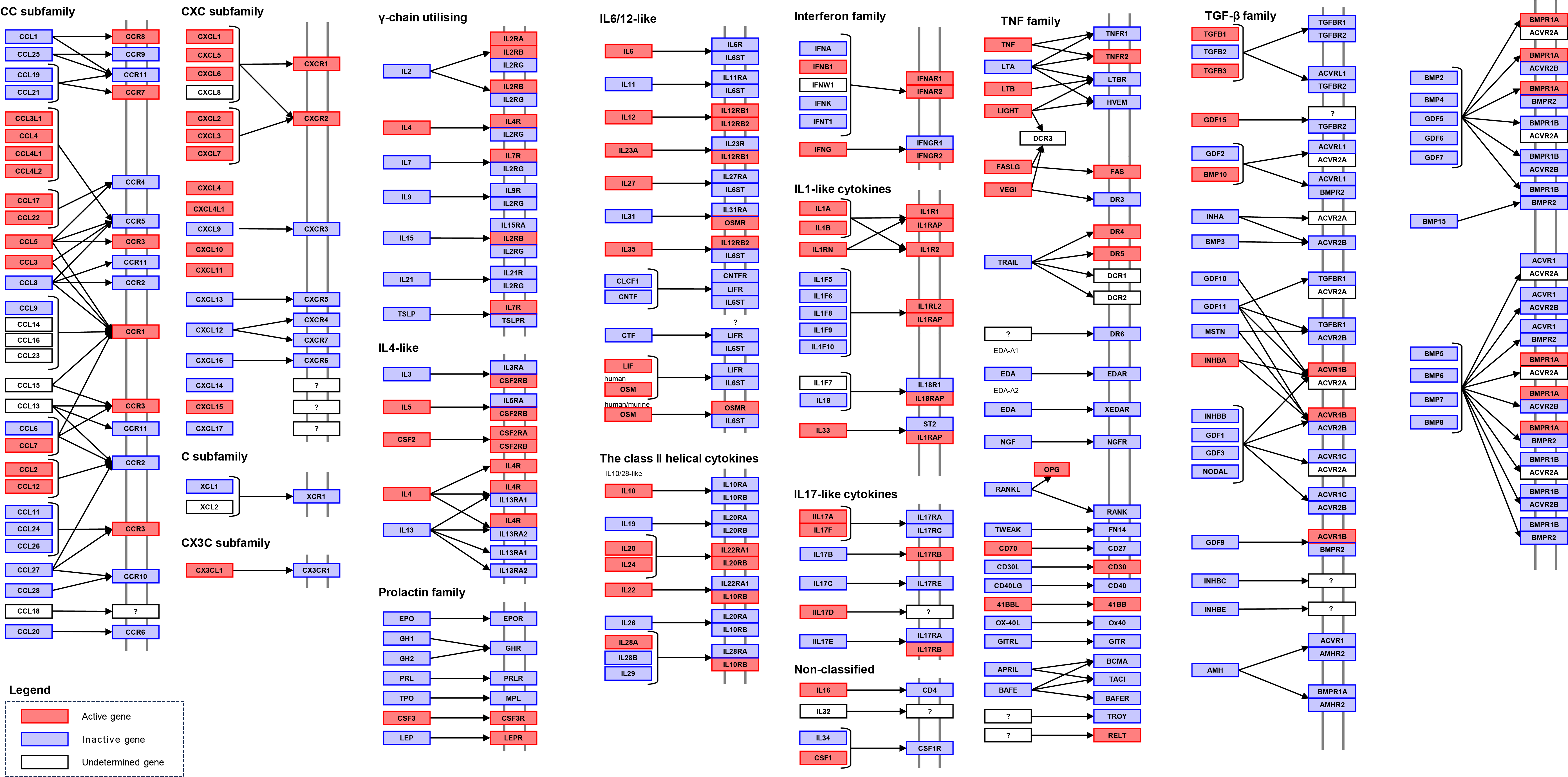

Supplement: Supplemental Material [file KVIR_A_2367659_SM5771.zip › Figure S3.png]

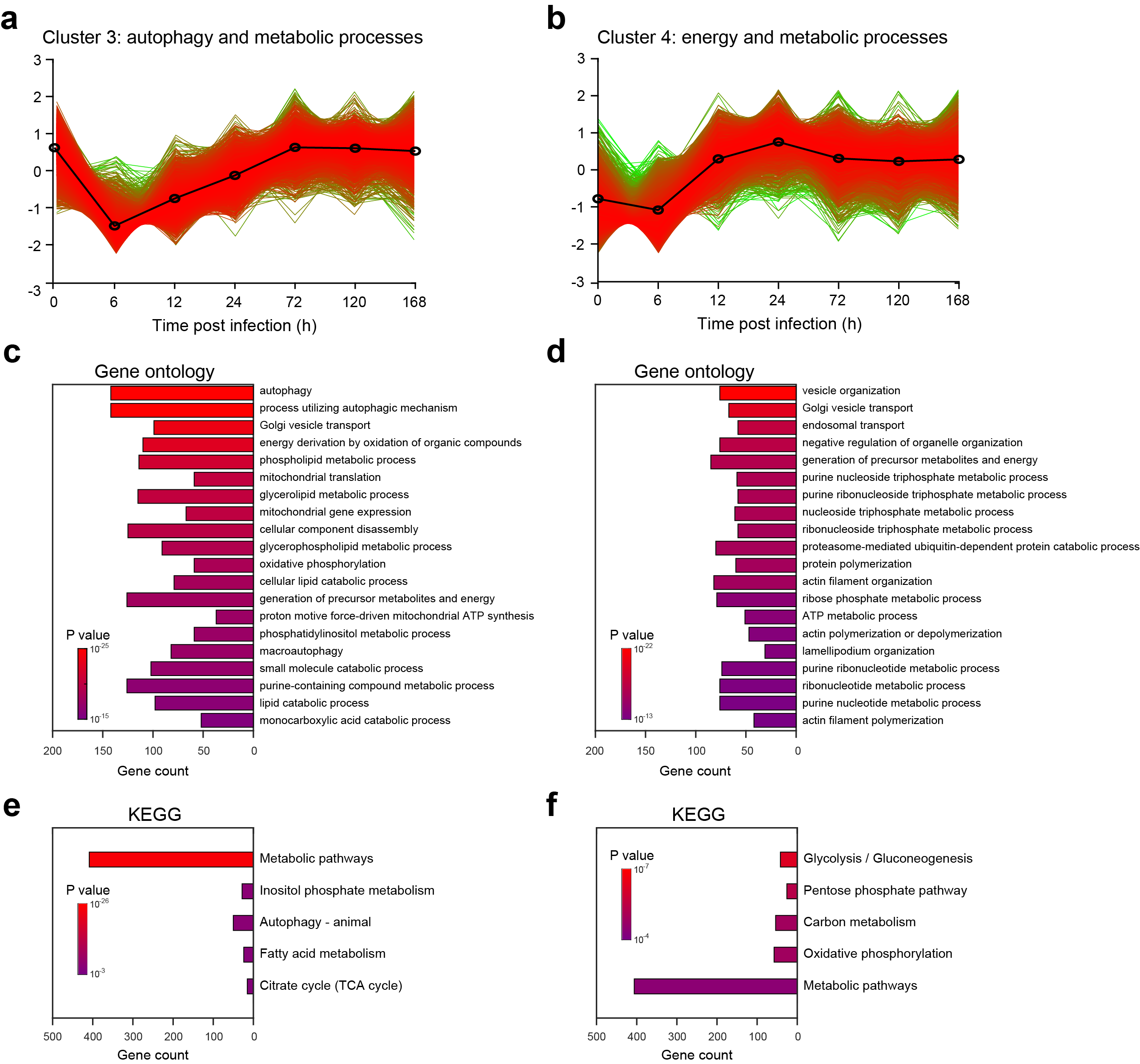

Supplement: Supplemental Material [file KVIR_A_2367659_SM5771.zip › Figure S4.png]

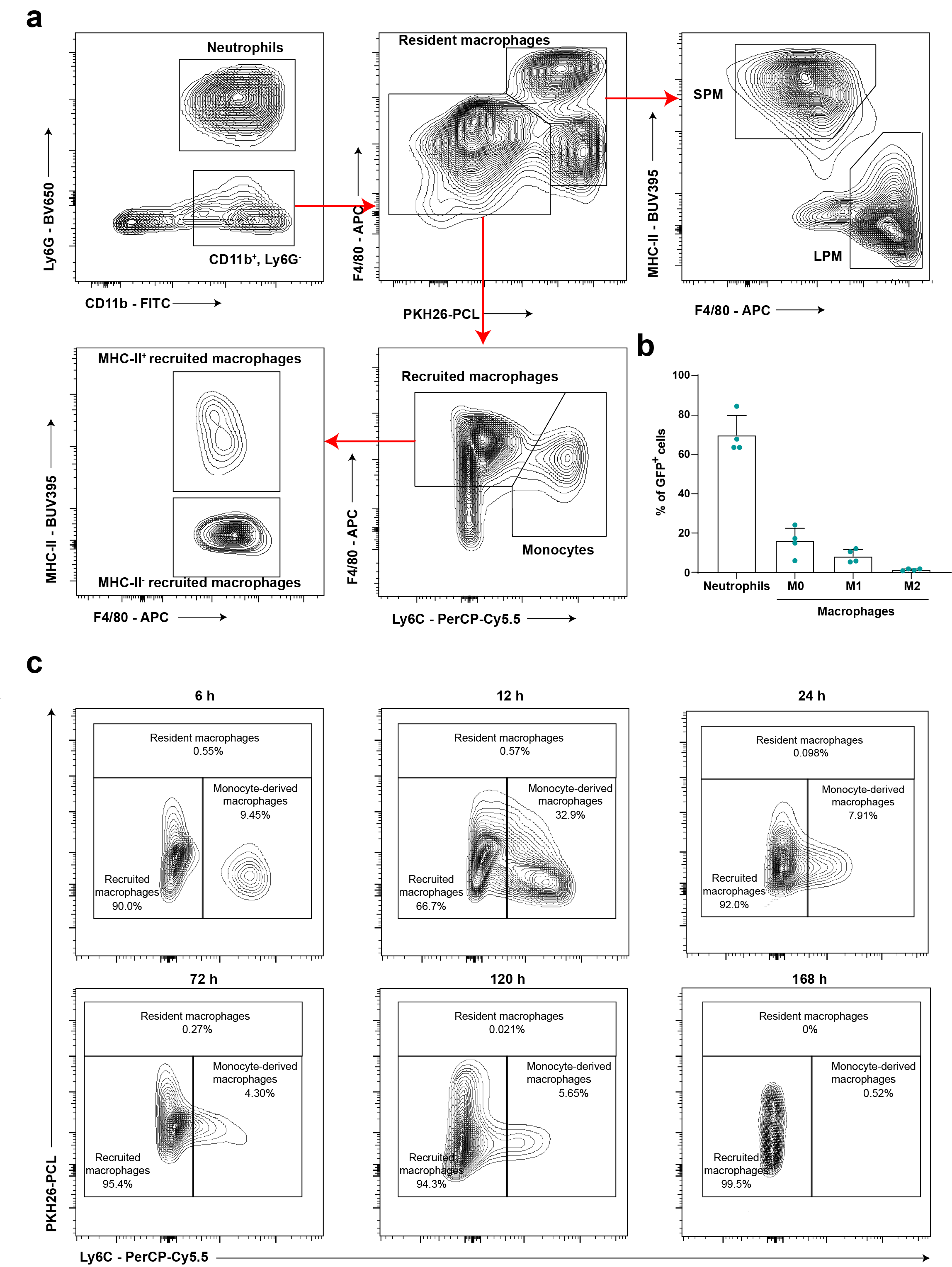

Supplement: Supplemental Material [file KVIR_A_2367659_SM5771.zip › Figure S5.png]

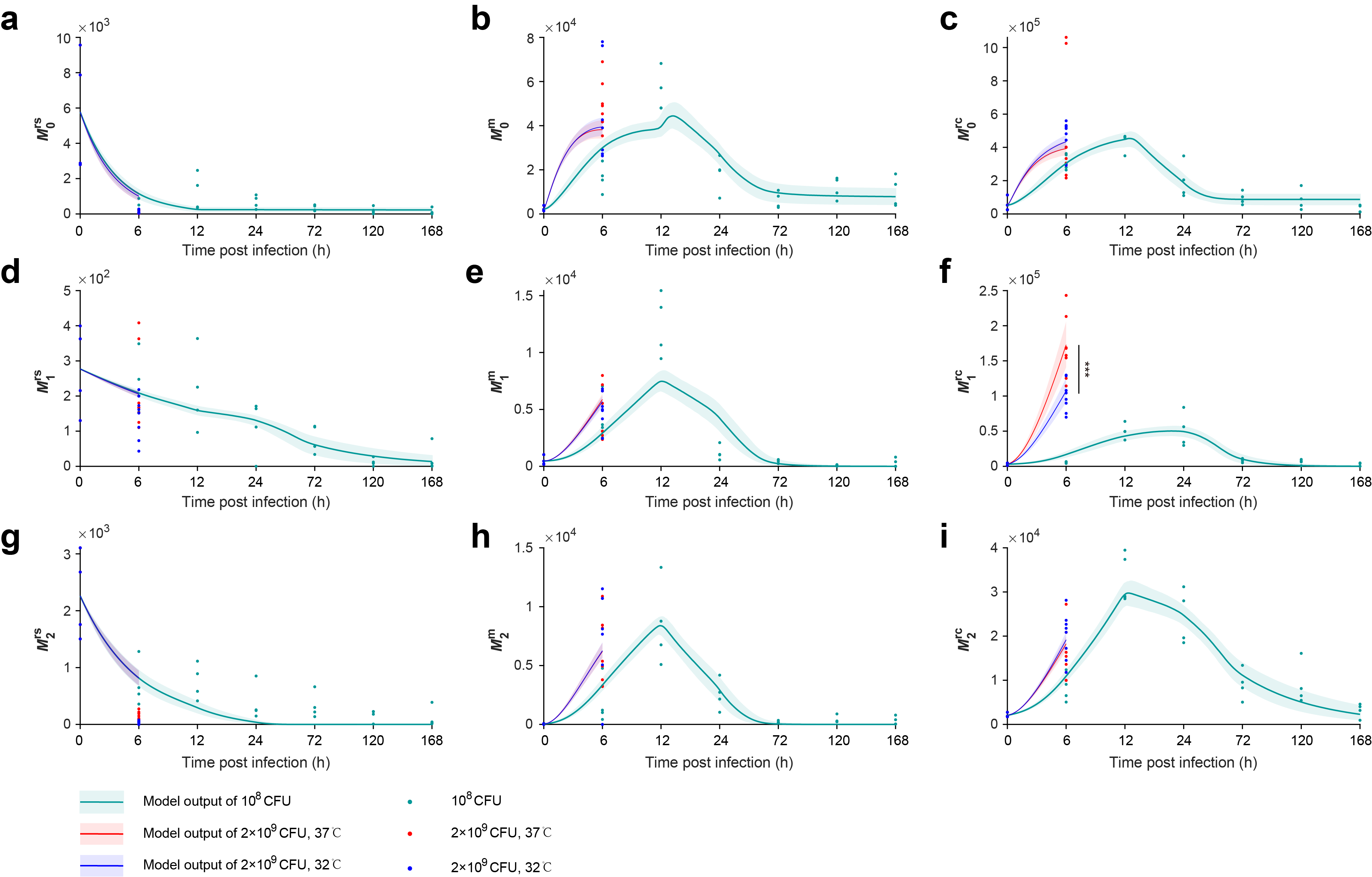

Supplement: Supplemental Material [file KVIR_A_2367659_SM5771.zip › Figure S6.png]
